# Supplementary figures and images for: A crosstalk between hepcidin and IRE/IRP pathways controls ferroportin expression and determines serum iron levels in mice
Source: eLife. 2022 Sep 6;11:e81332. doi: 10.7554/eLife.81332 (PMC9499557; doi:10.7554/eLife.81332)

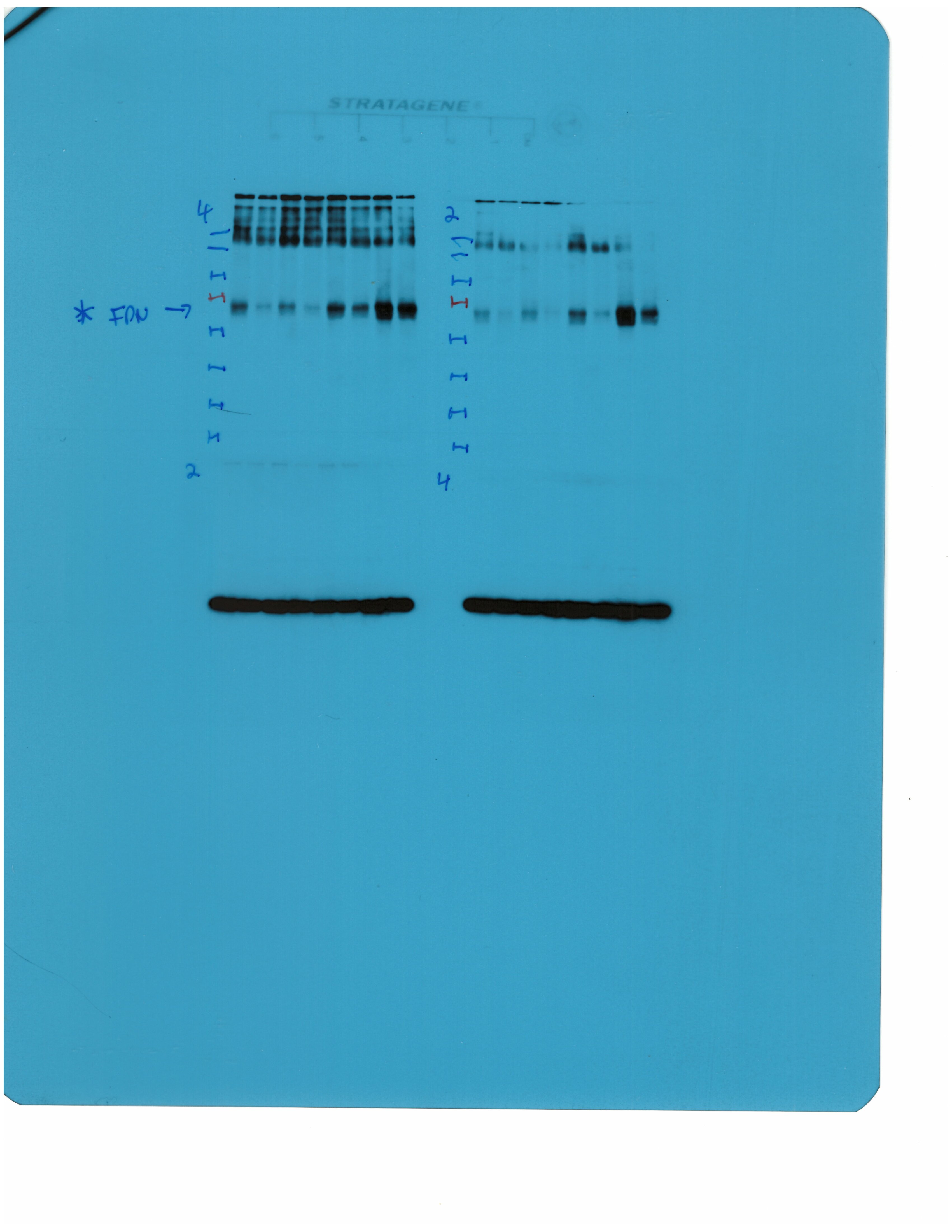

Supplement: Figure 3—source data 2. [file elife-81332-fig3-data2.jpg]

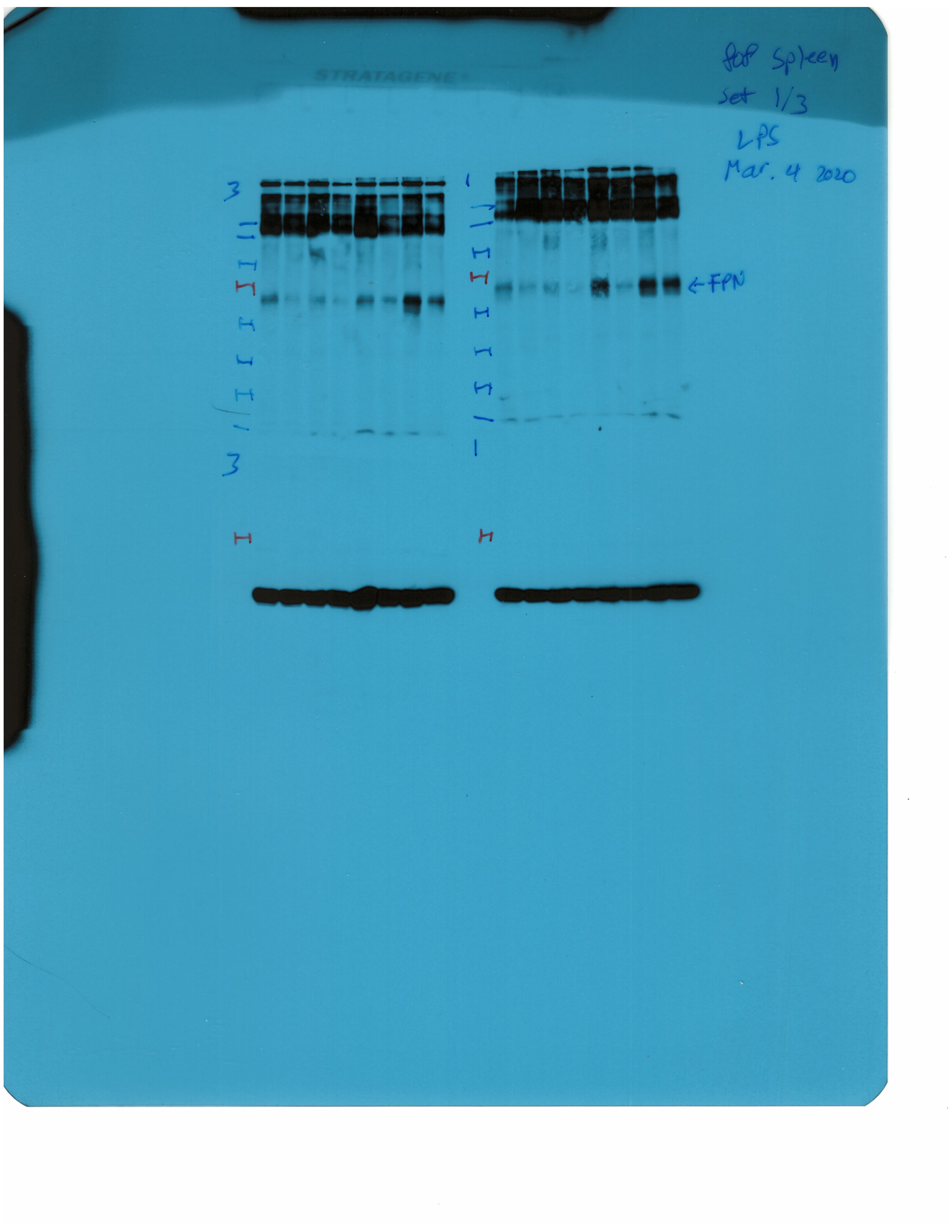

Supplement: Figure 3—source data 3. [file elife-81332-fig3-data3.jpg]

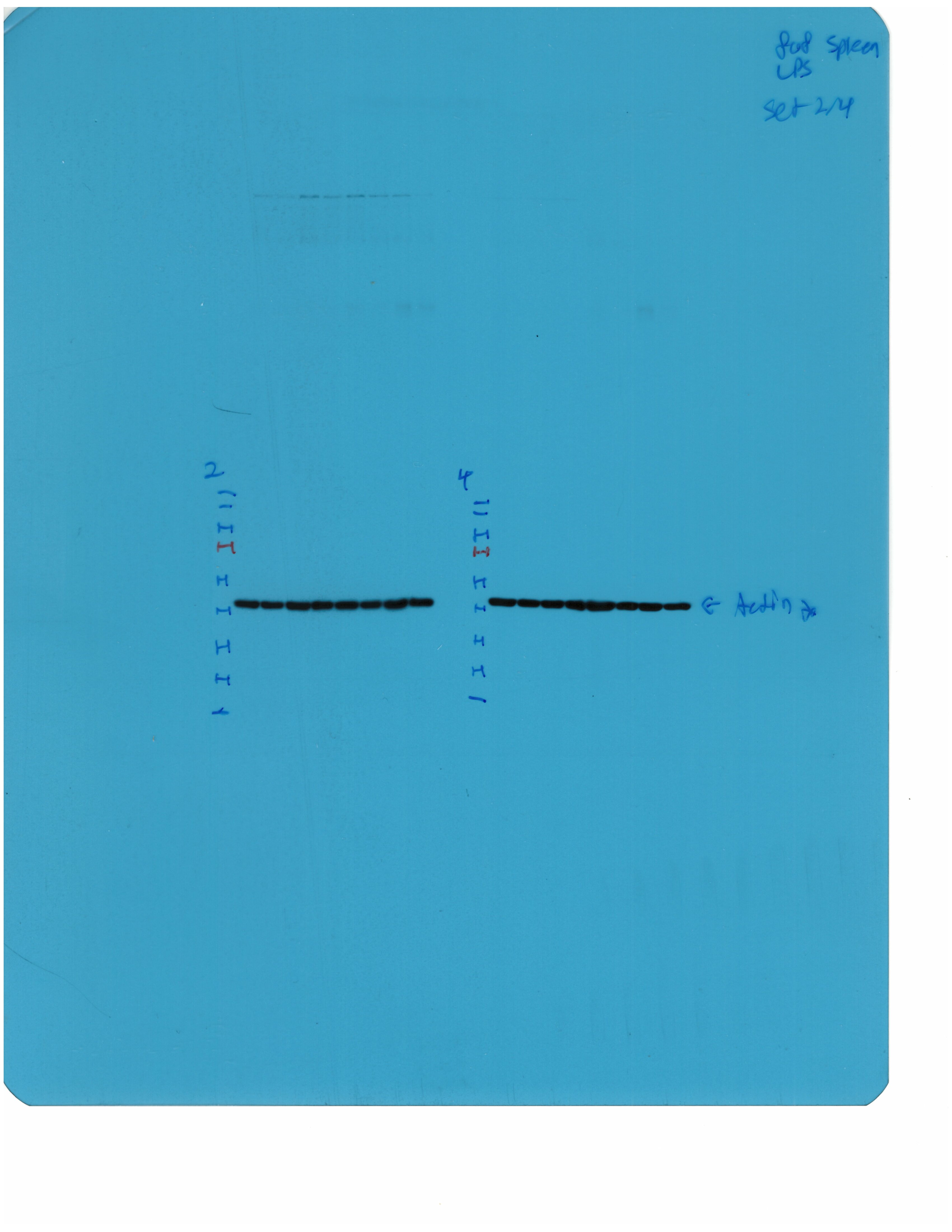

Supplement: Figure 3—source data 4. [file elife-81332-fig3-data4.jpg]

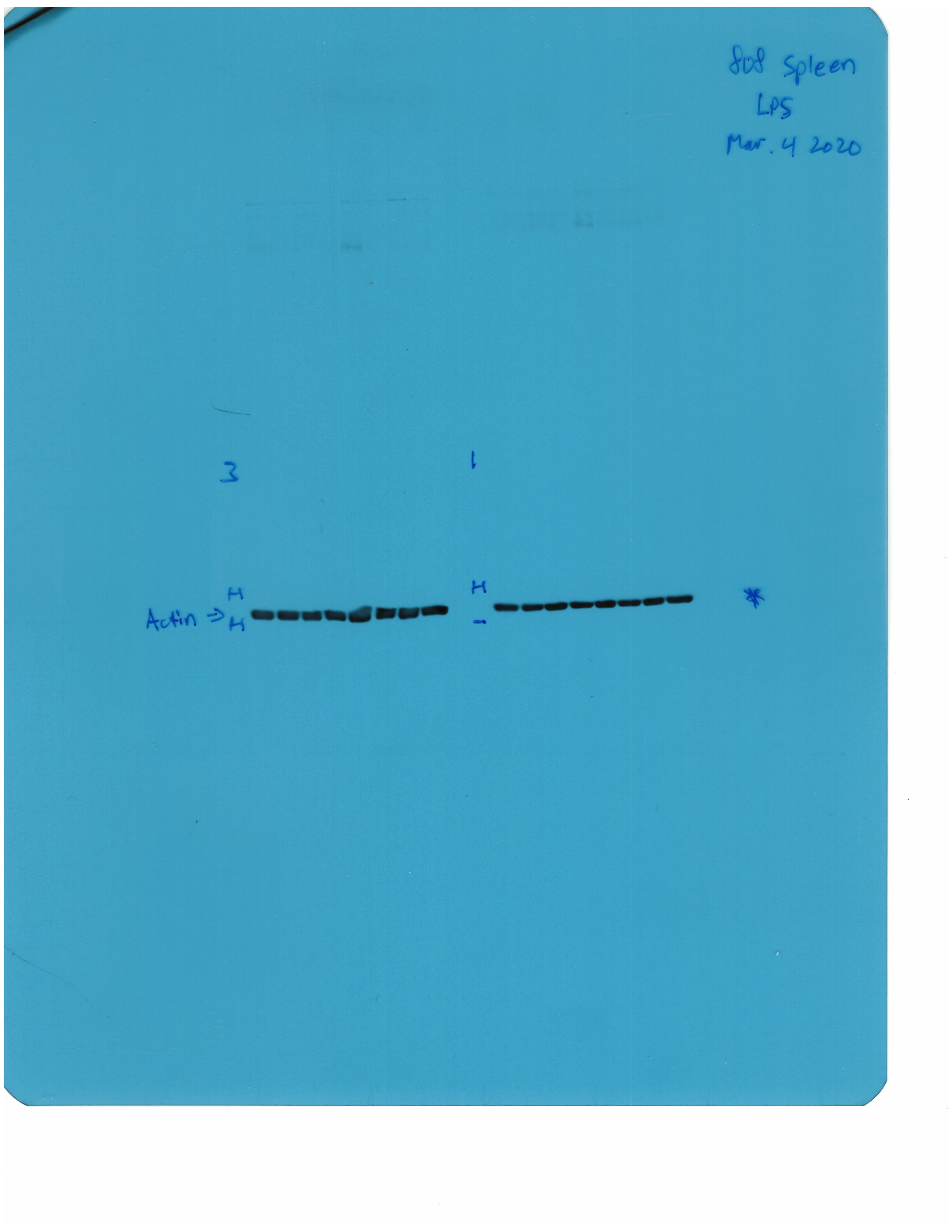

Supplement: Figure 3—source data 5. [file elife-81332-fig3-data5.jpg]

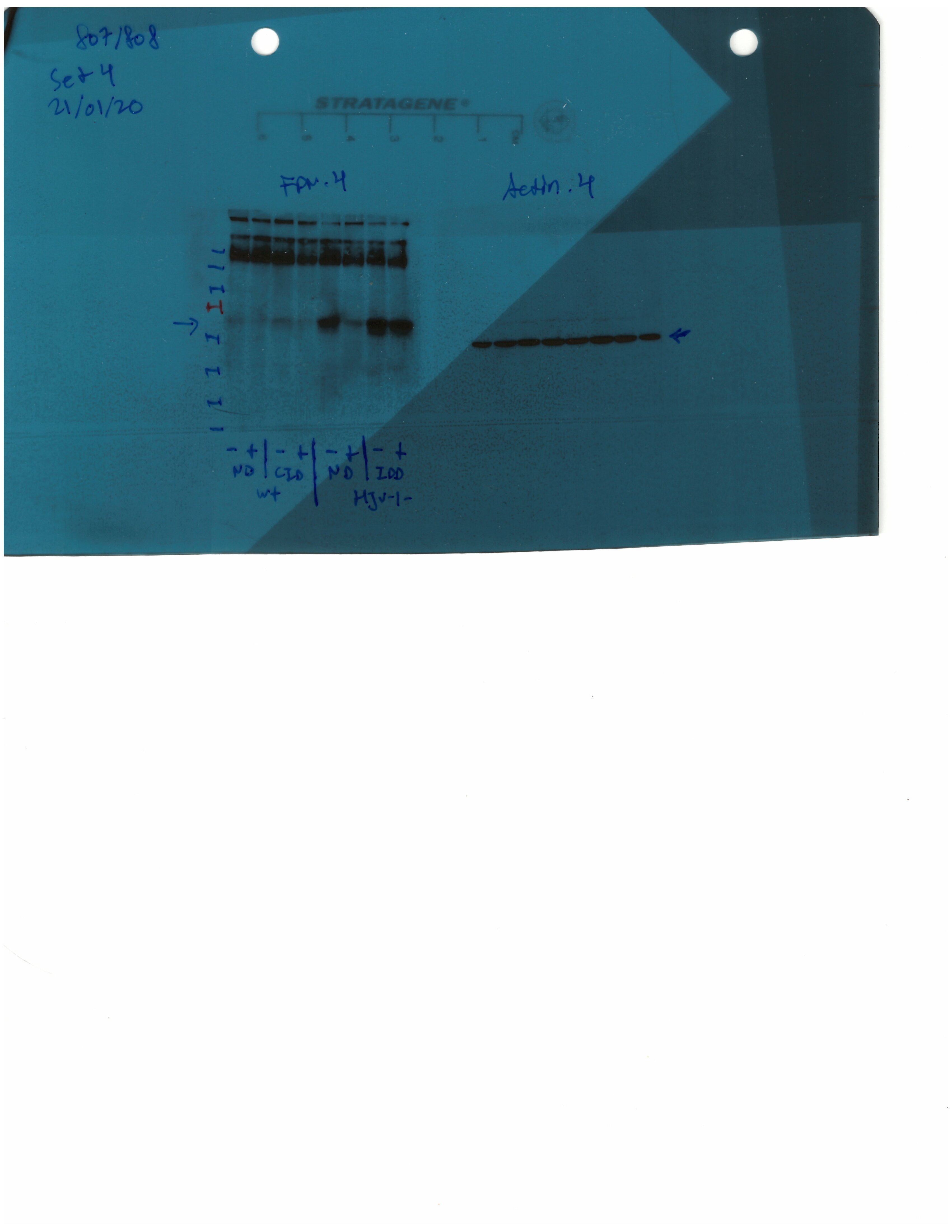

Supplement: Figure 3—source data 6. [file elife-81332-fig3-data6.jpg]

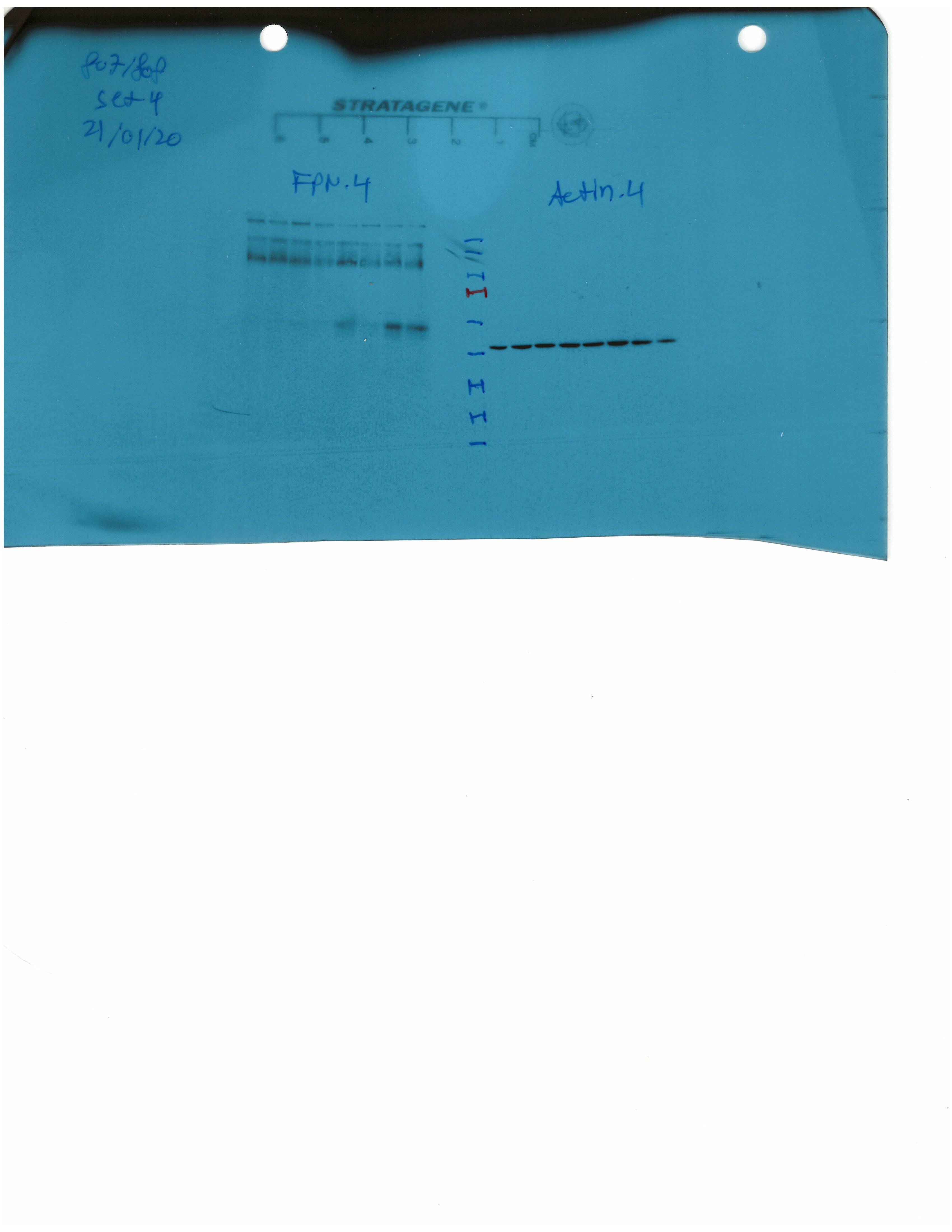

Supplement: Figure 3—source data 7. [file elife-81332-fig3-data7.jpg]

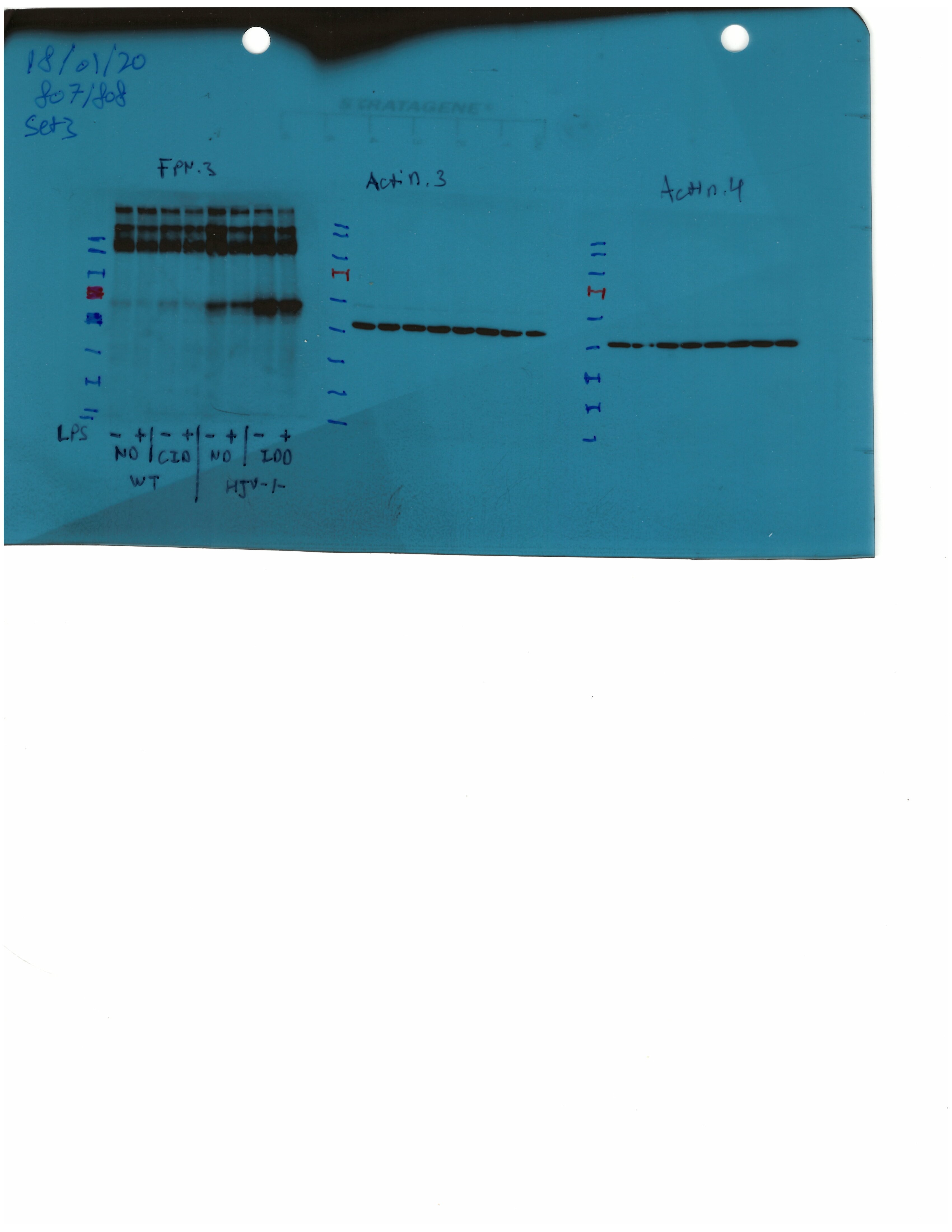

Supplement: Figure 3—source data 8. [file elife-81332-fig3-data8.jpg]

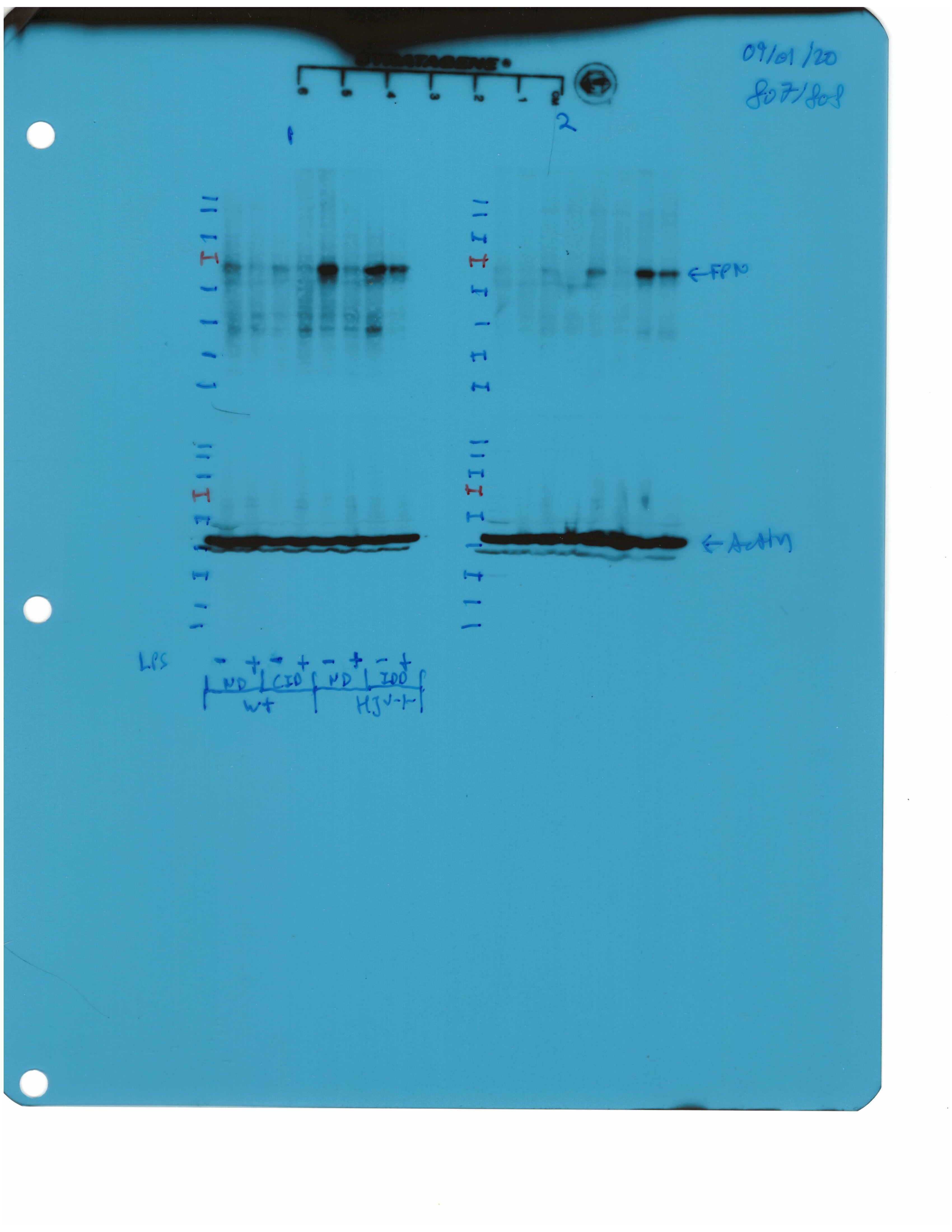

Supplement: Figure 3—source data 9. [file elife-81332-fig3-data9.jpg]

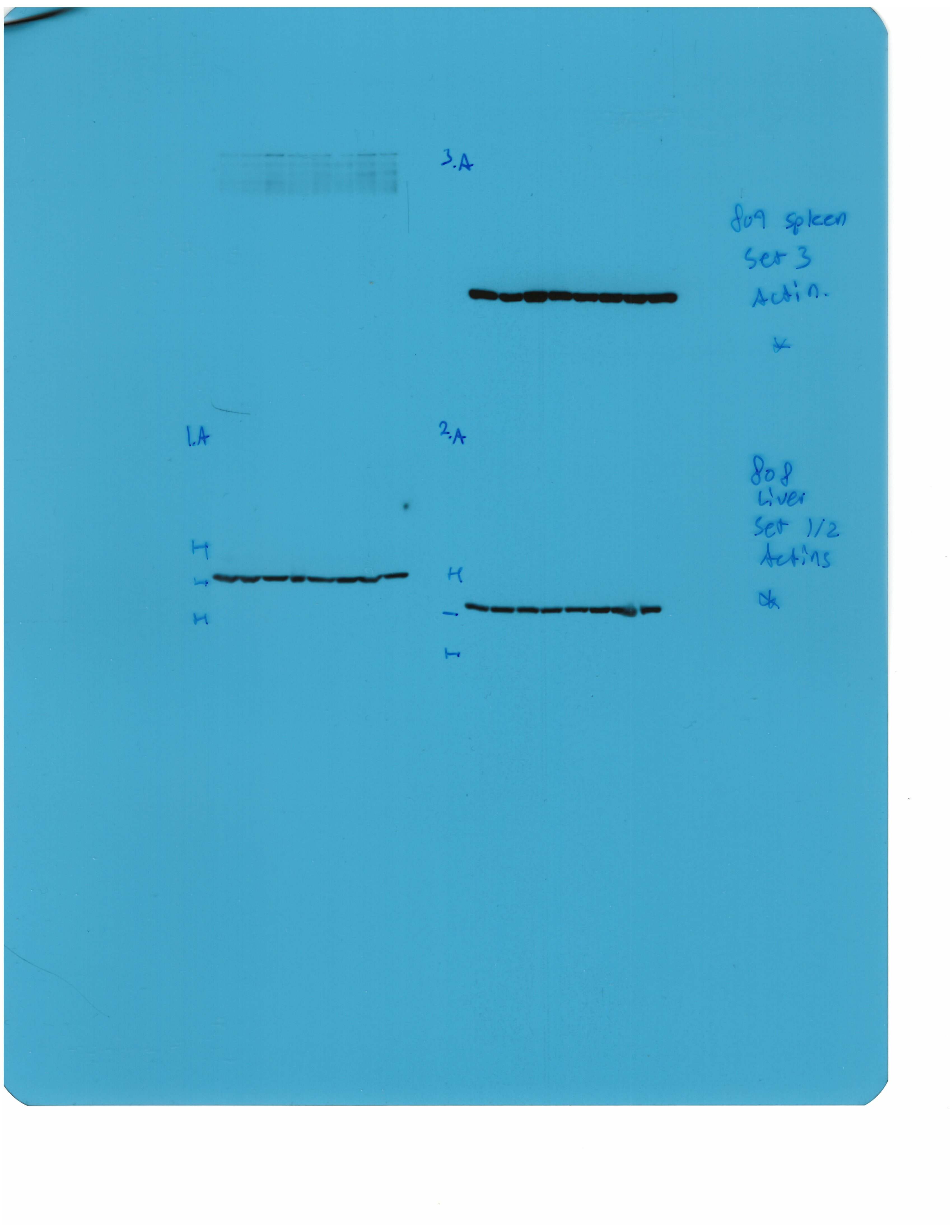

Supplement: Figure 3—source data 10. [file elife-81332-fig3-data10.jpg]

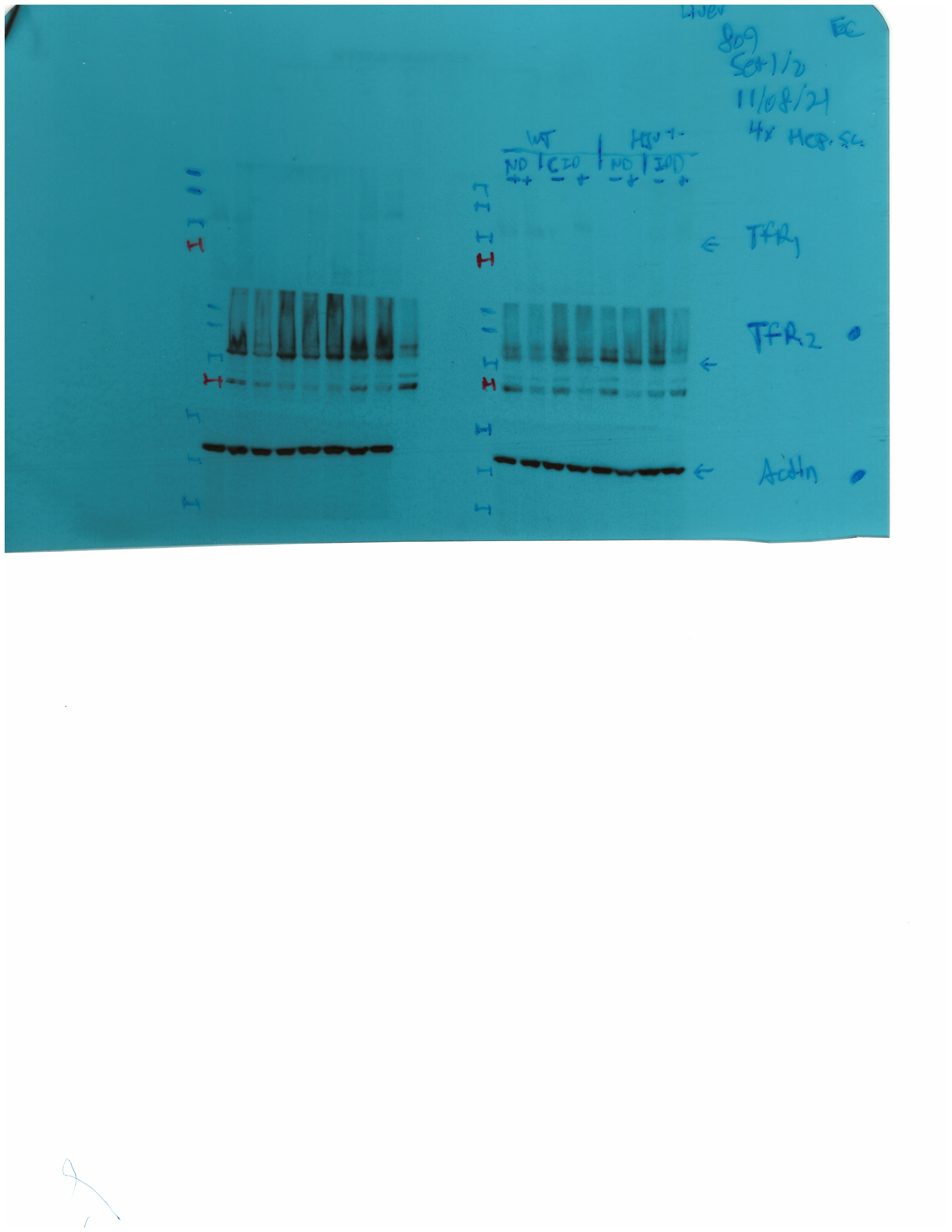

Supplement: Figure 4—figure supplement 2—source data 2. [file elife-81332-fig4-figsupp2-data2.jpg]

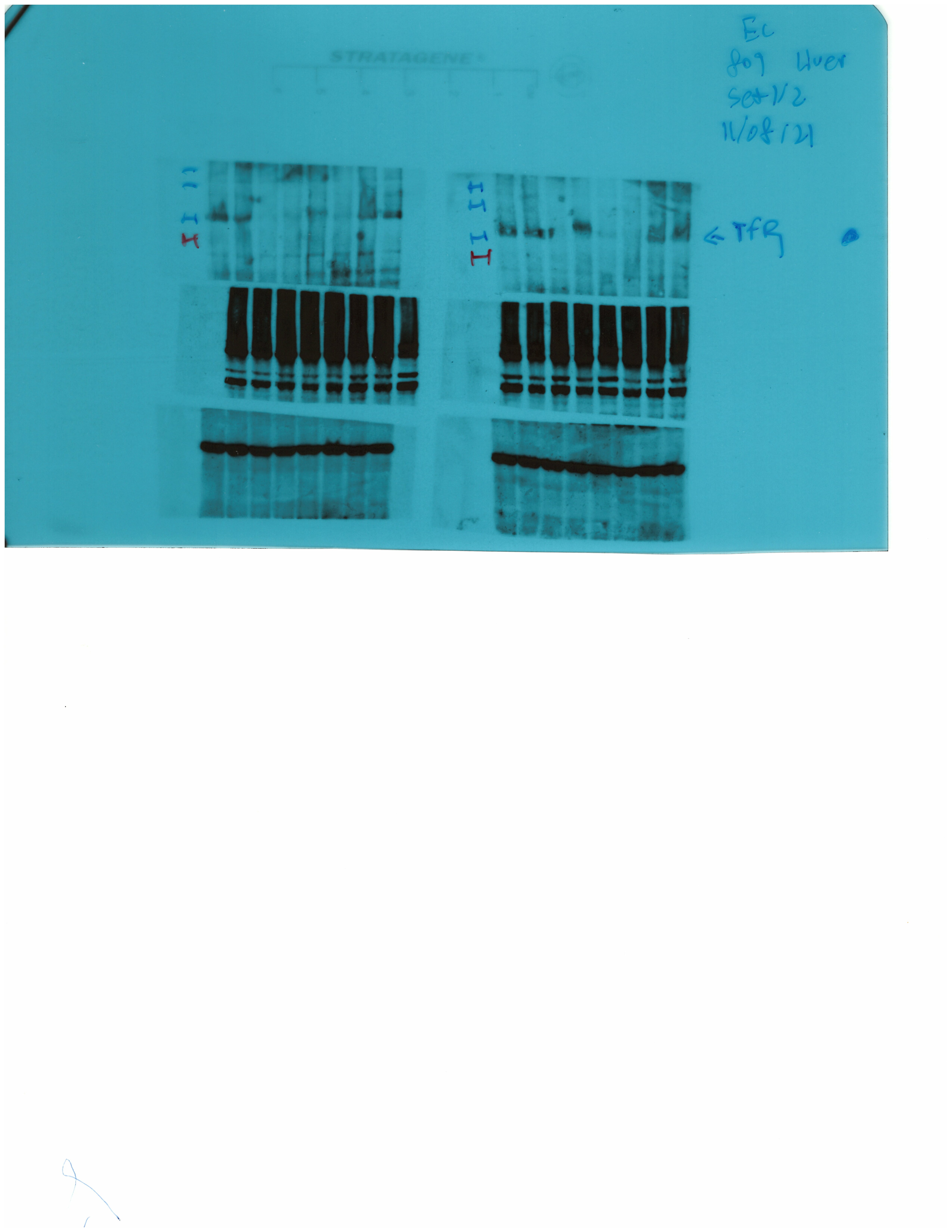

Supplement: Figure 4—figure supplement 2—source data 3. [file elife-81332-fig4-figsupp2-data3.jpg]

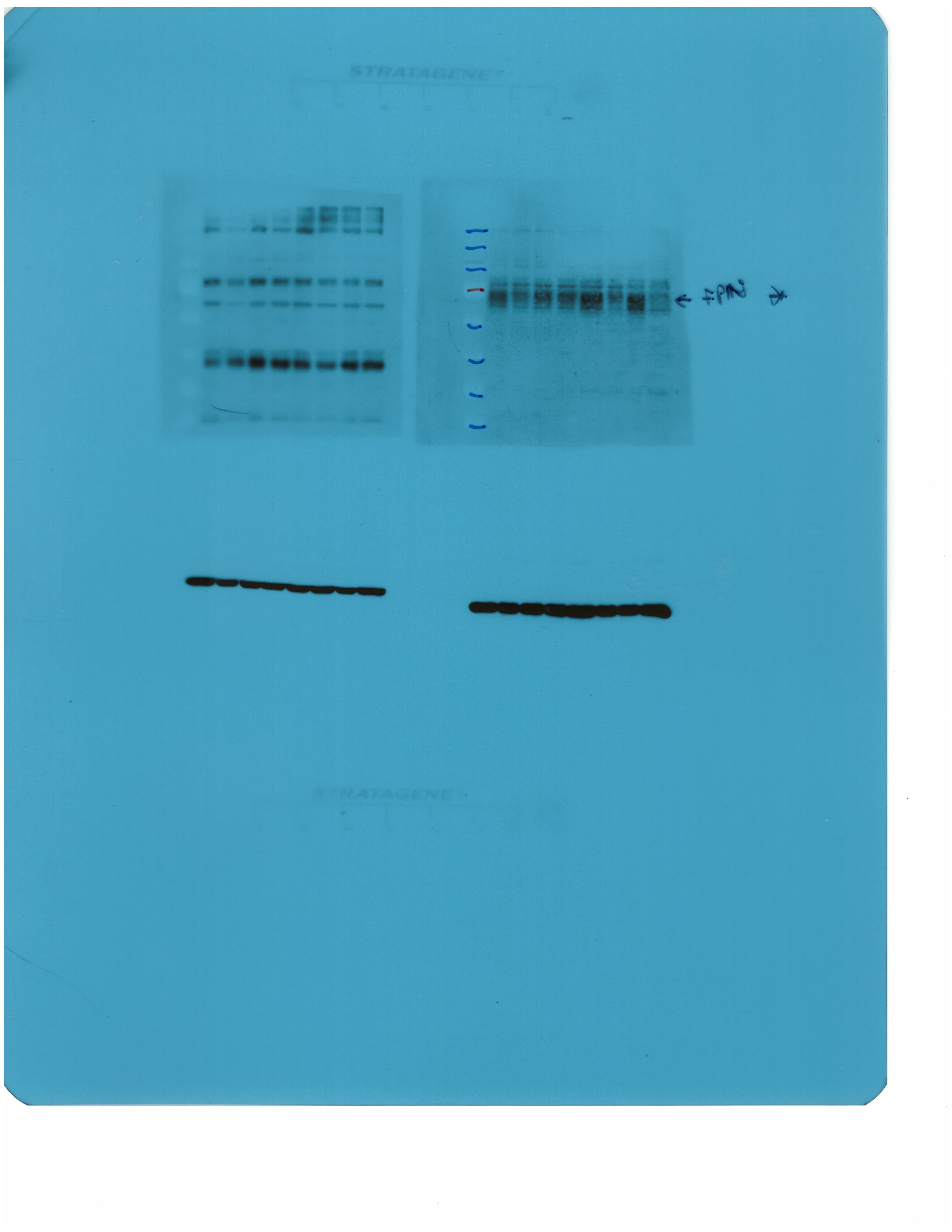

Supplement: Figure 5—source data 2. [file elife-81332-fig5-data2.jpg]

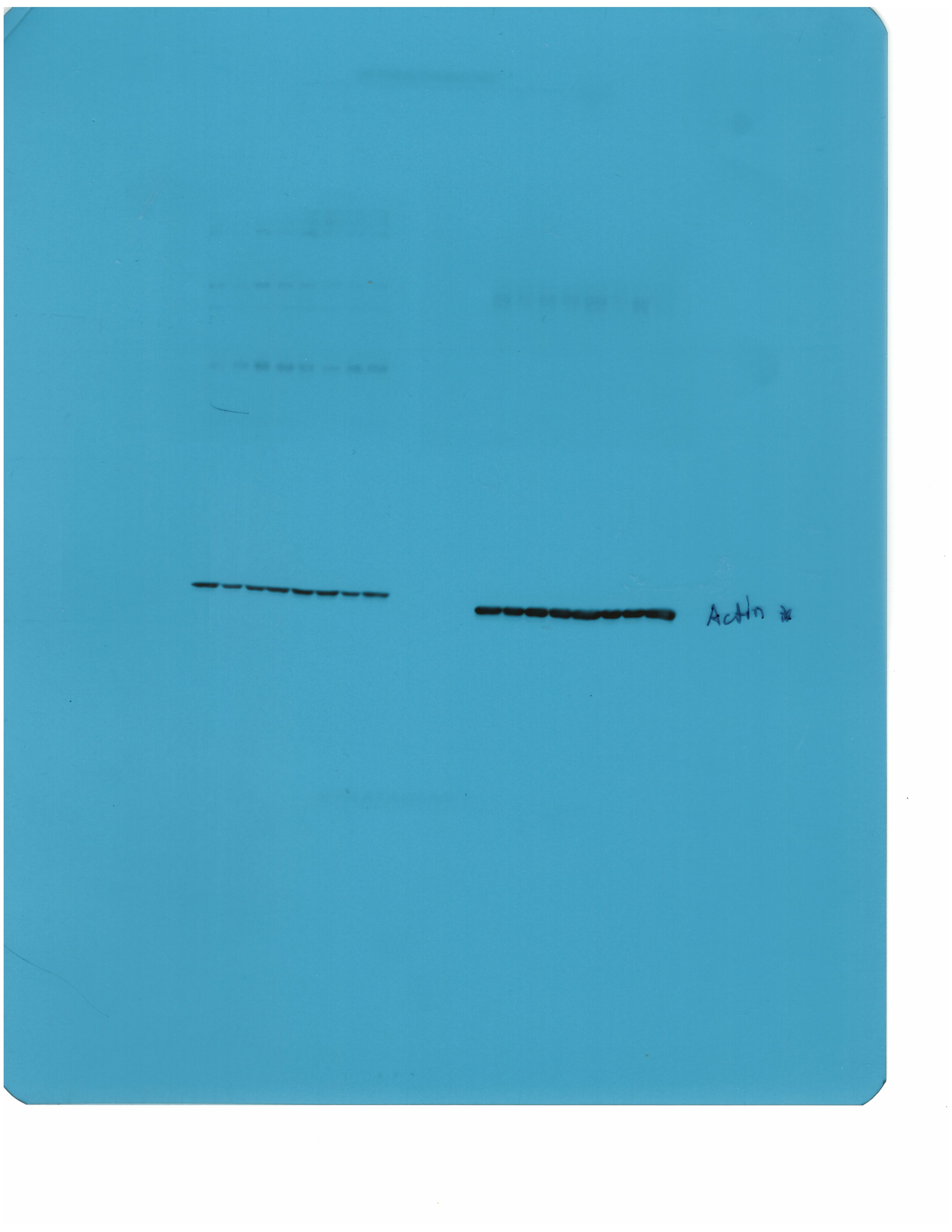

Supplement: Figure 5—source data 3. [file elife-81332-fig5-data3.jpg]

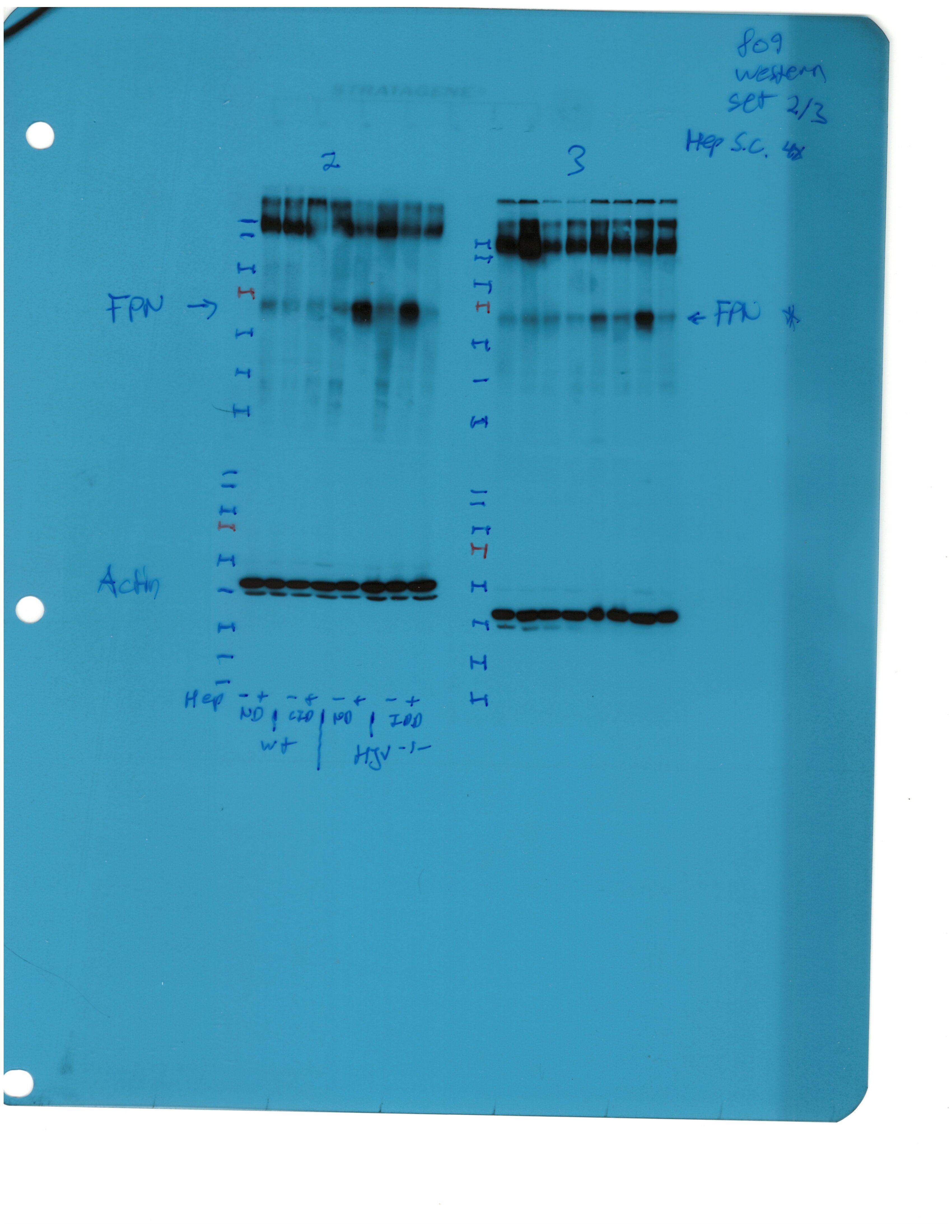

Supplement: Figure 5—source data 4. [file elife-81332-fig5-data4.jpg]

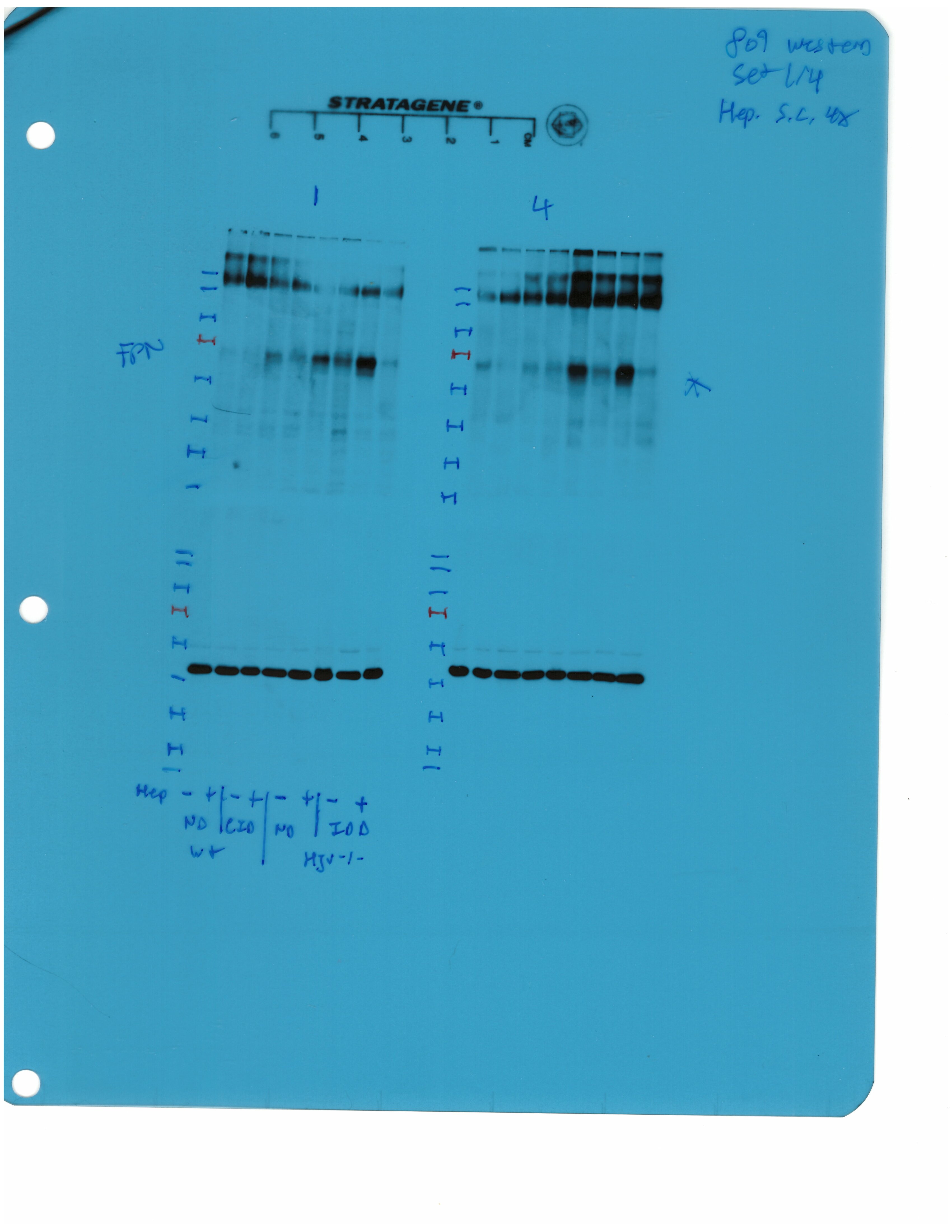

Supplement: Figure 5—source data 5. [file elife-81332-fig5-data5.jpg]

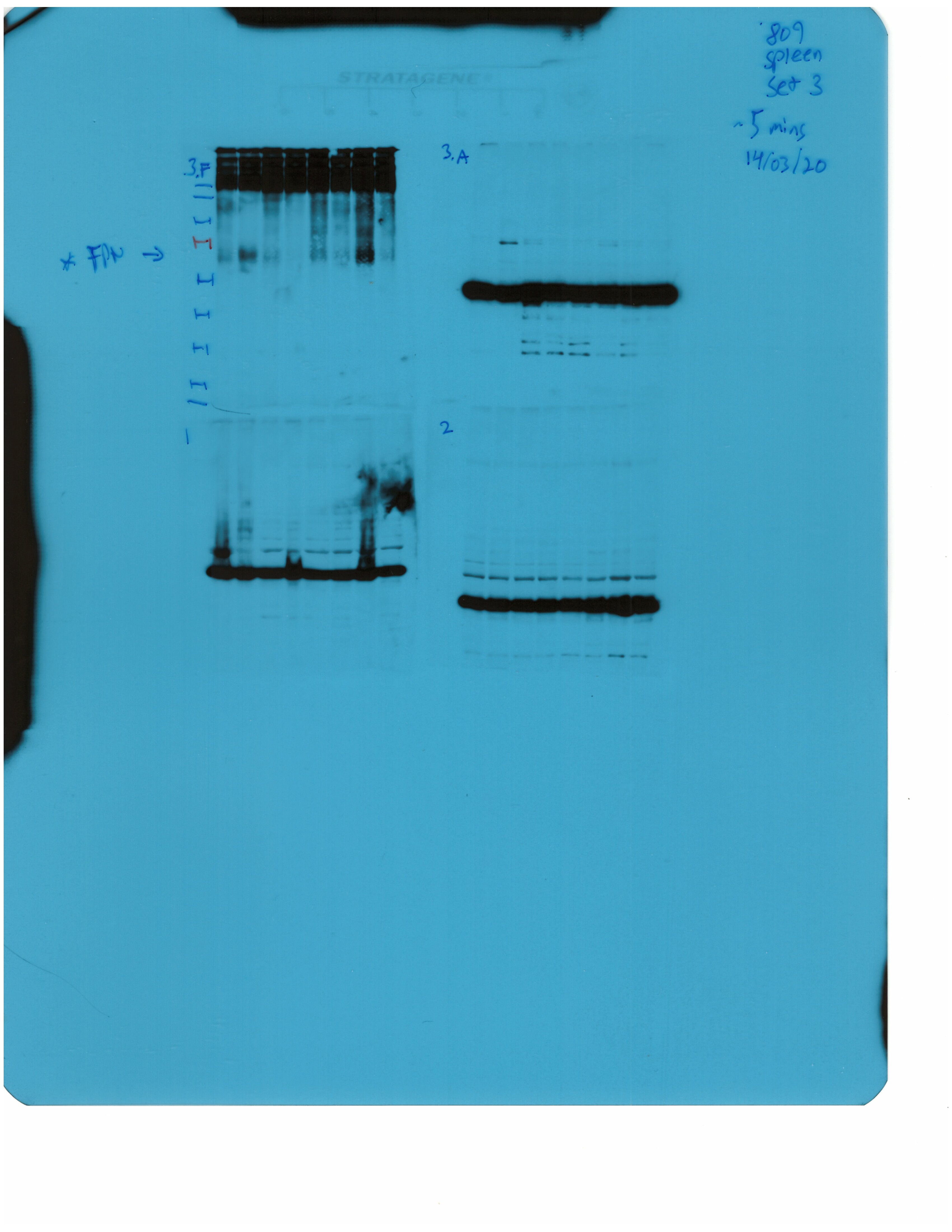

Supplement: Figure 5—source data 6. [file elife-81332-fig5-data6.jpg]

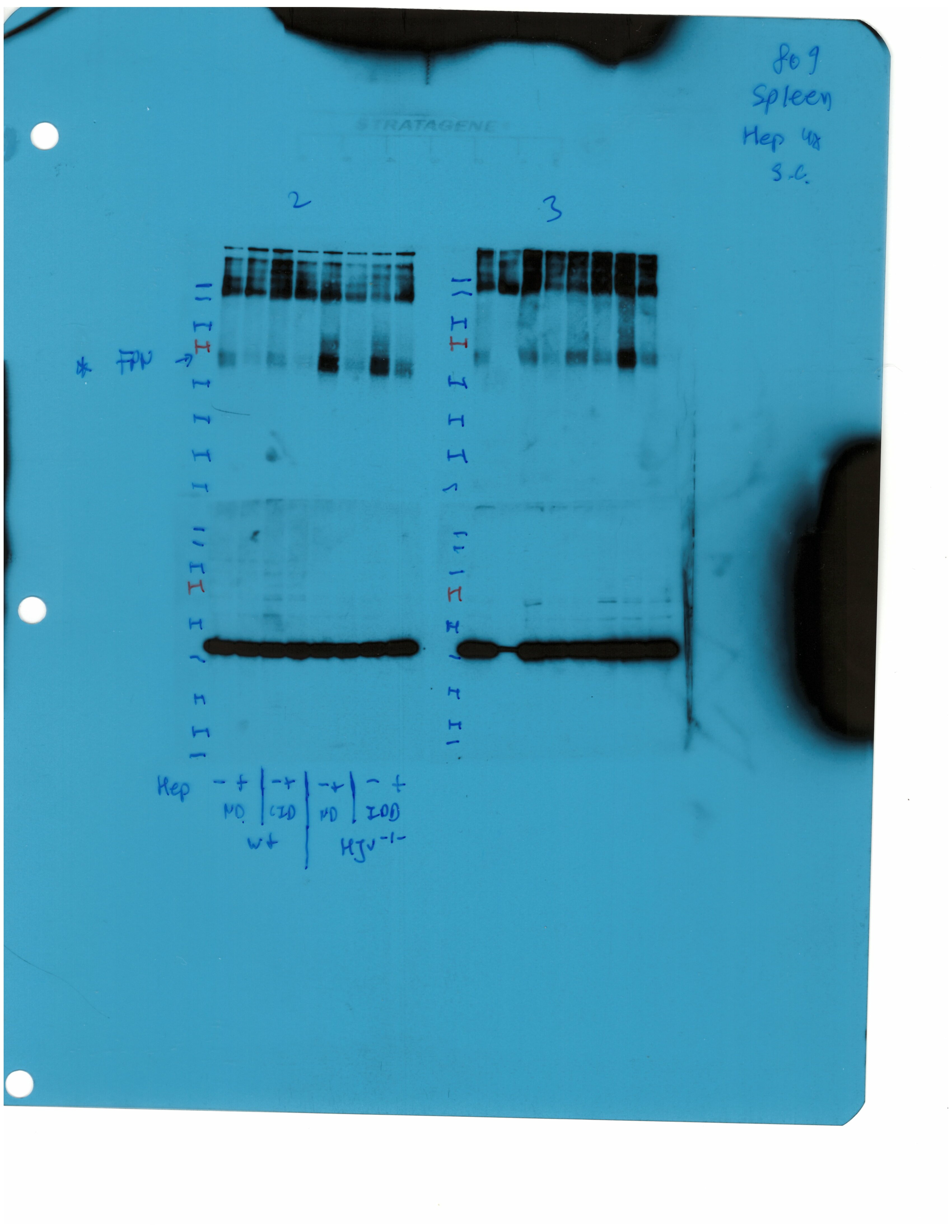

Supplement: Figure 5—source data 8. [file elife-81332-fig5-data8.jpg]

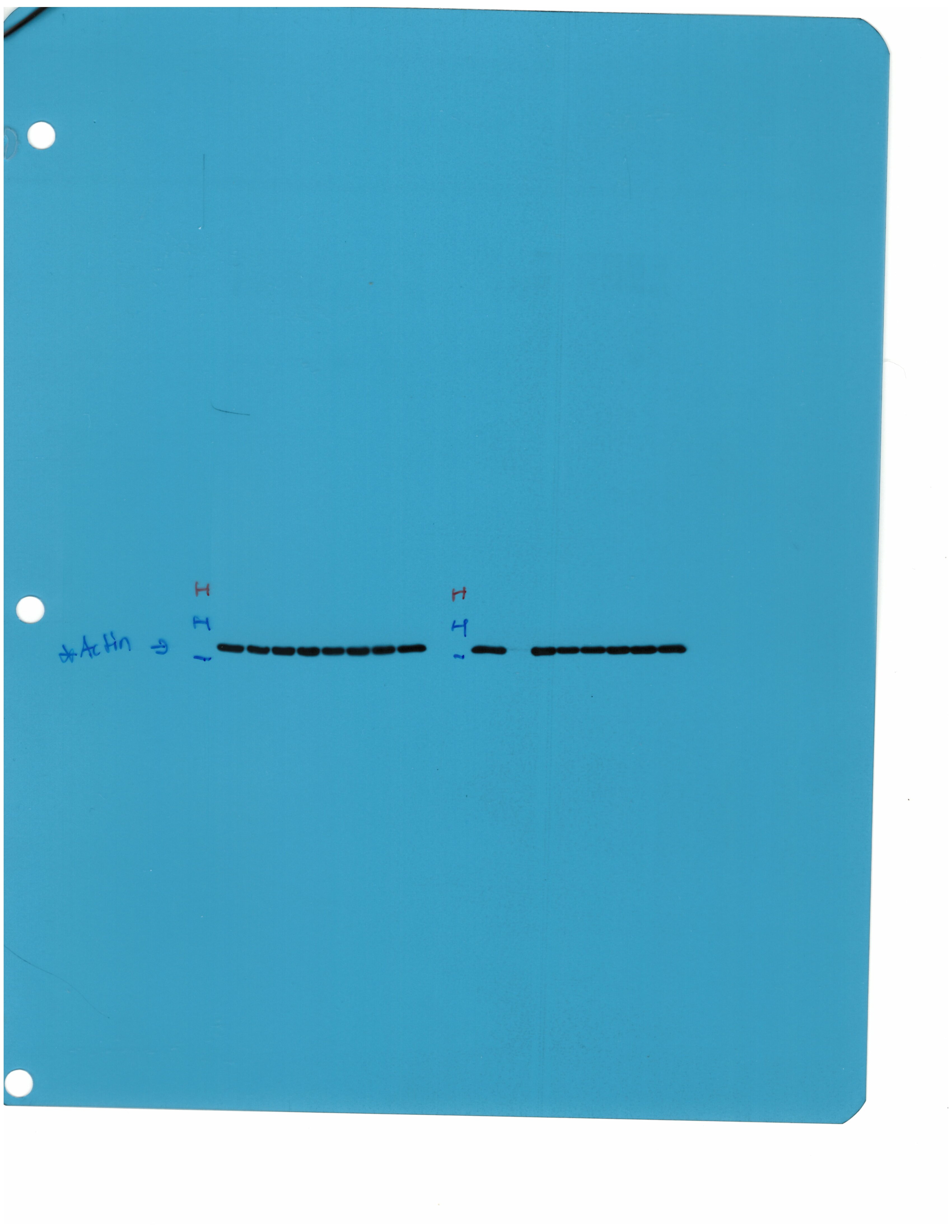

Supplement: Figure 5—source data 9. [file elife-81332-fig5-data9.jpg]

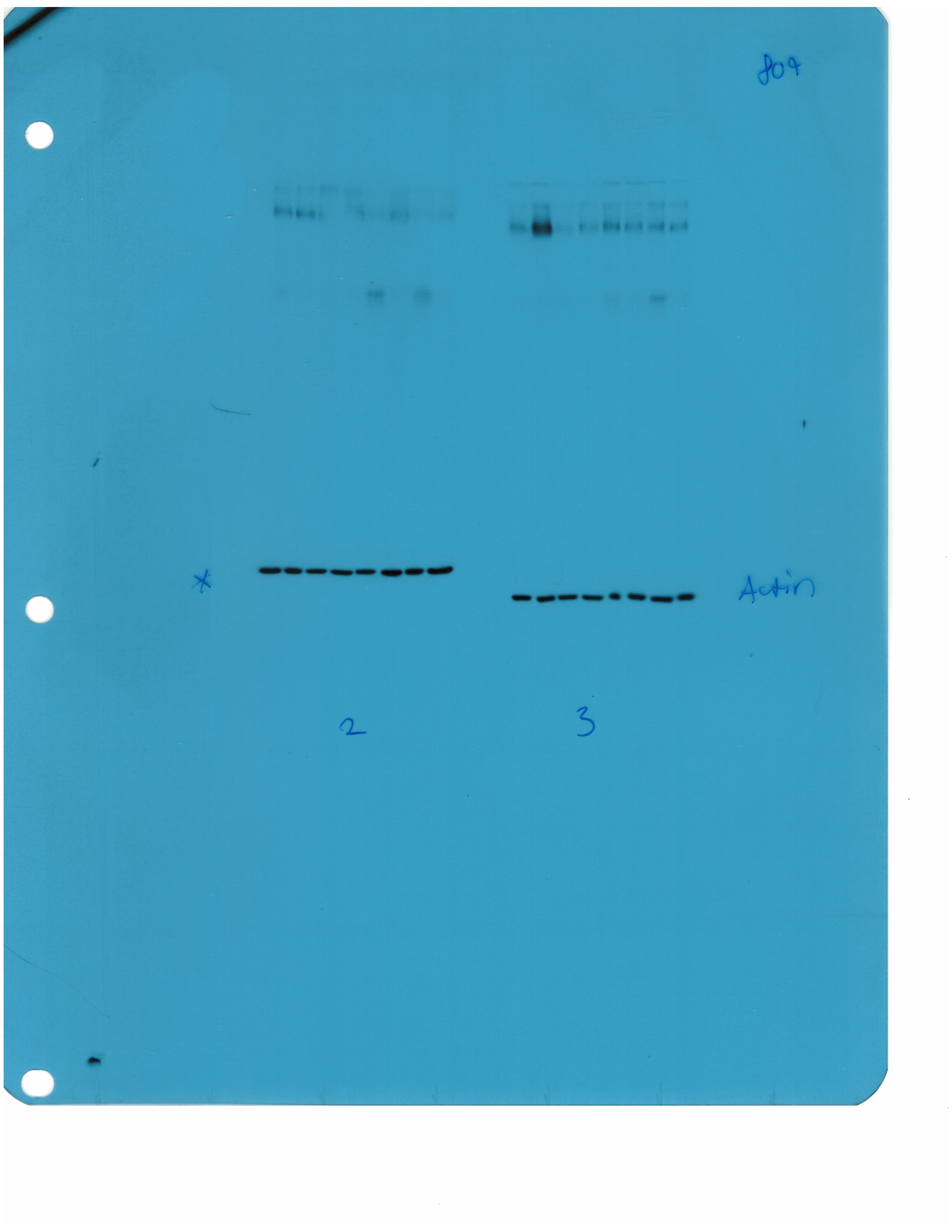

Supplement: Figure 5—source data 10. [file elife-81332-fig5-data10.jpg]

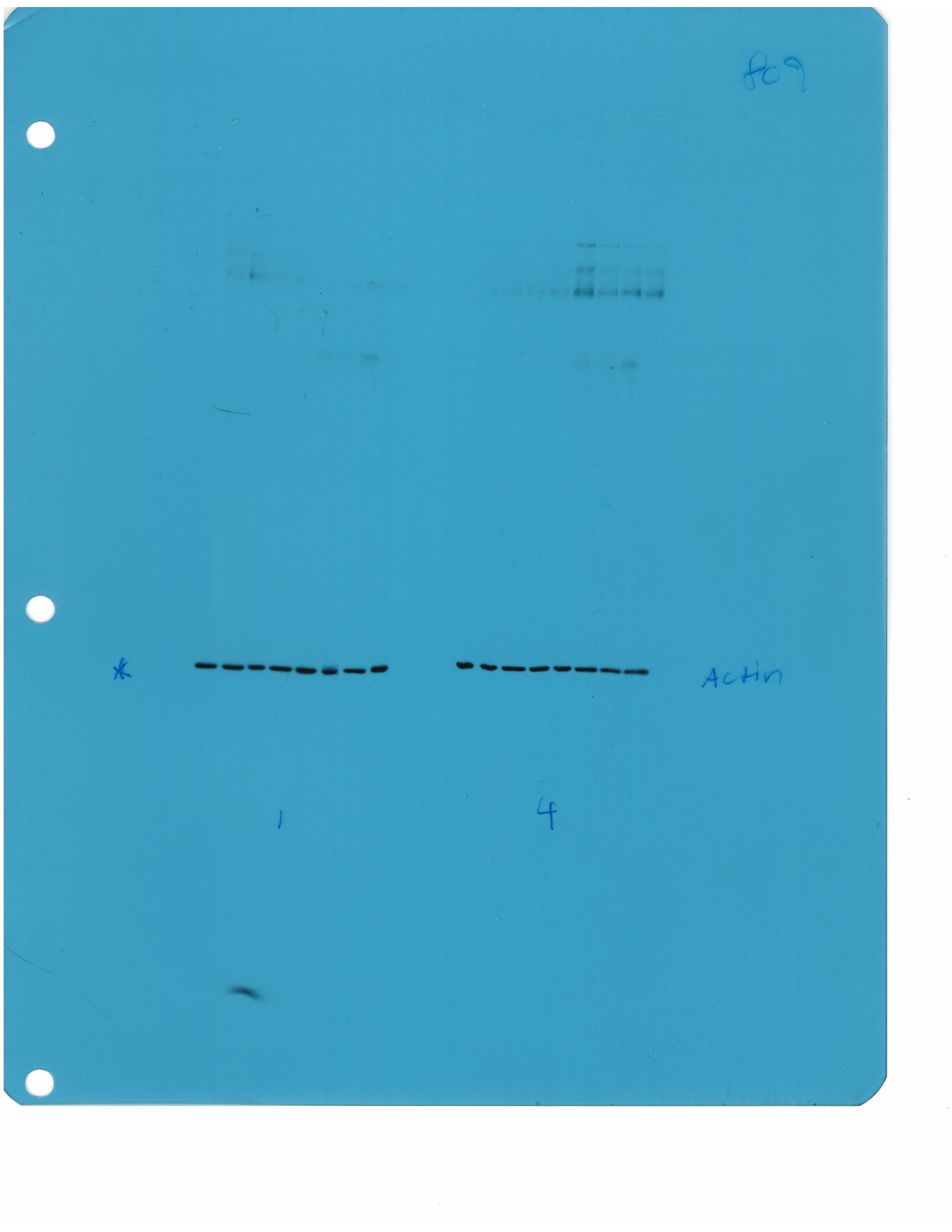

Supplement: Figure 5—source data 11. [file elife-81332-fig5-data11.jpg]

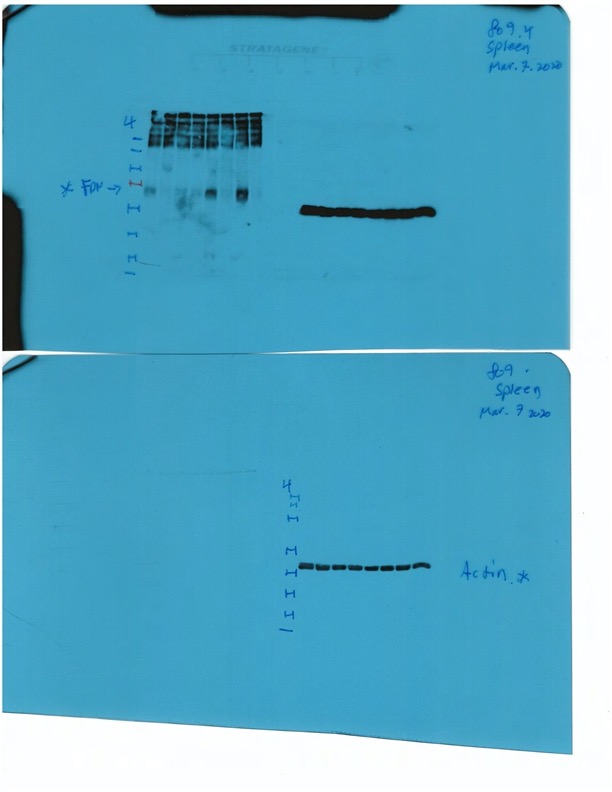

Supplement: Figure 5—source data 23. [file elife-81332-fig5-data23.jpg]

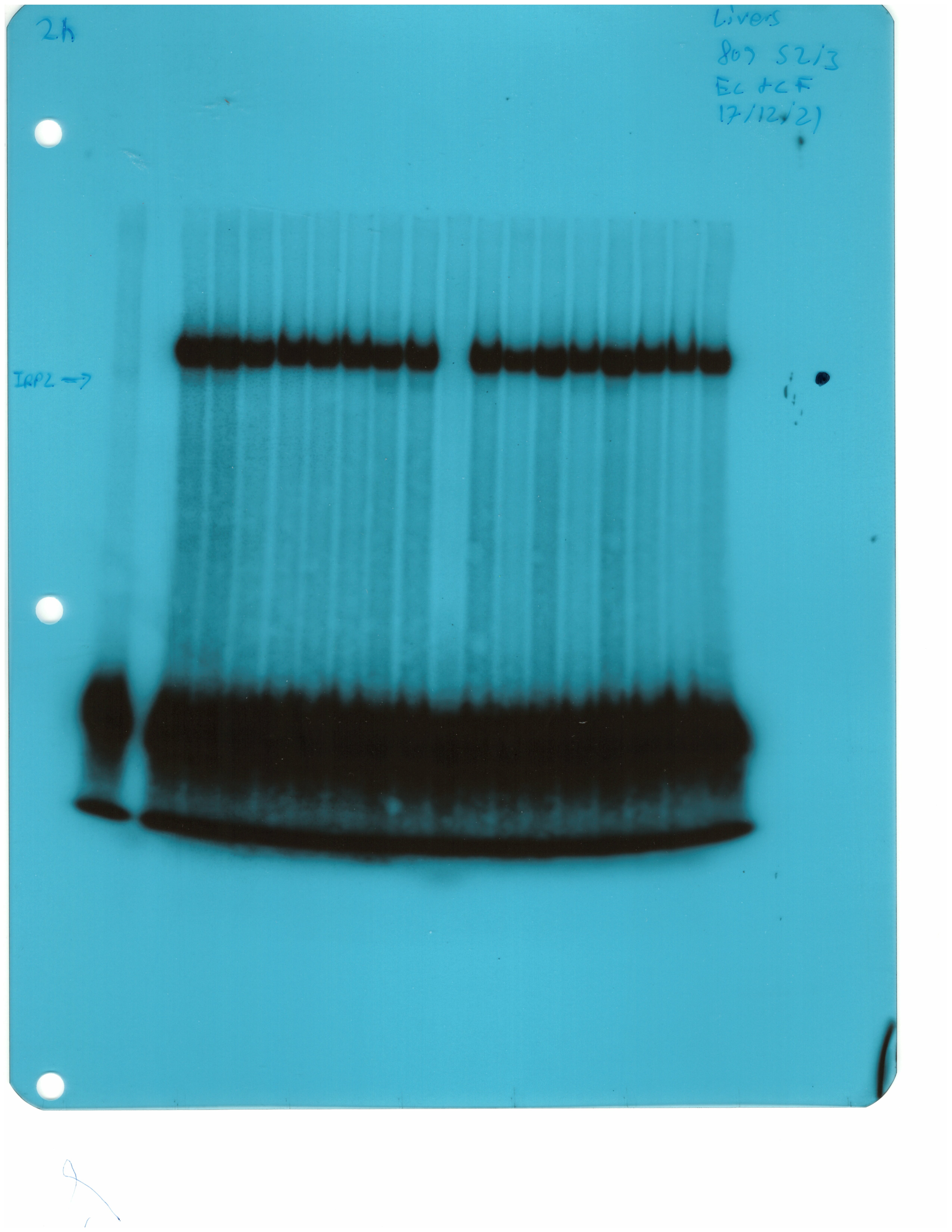

Supplement: Figure 6—source data 3. [file elife-81332-fig6-data3.jpg]

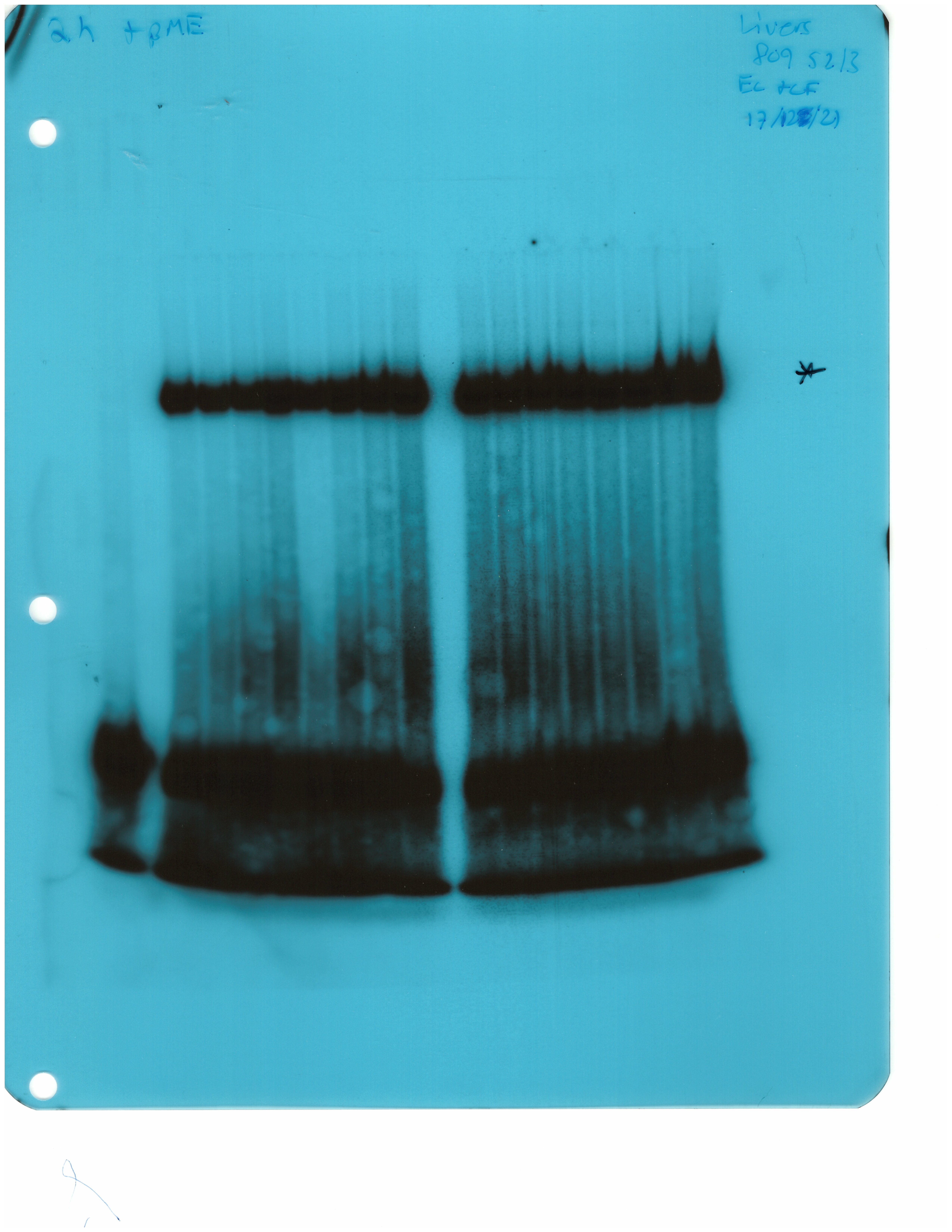

Supplement: Figure 6—source data 4. [file elife-81332-fig6-data4.jpg]

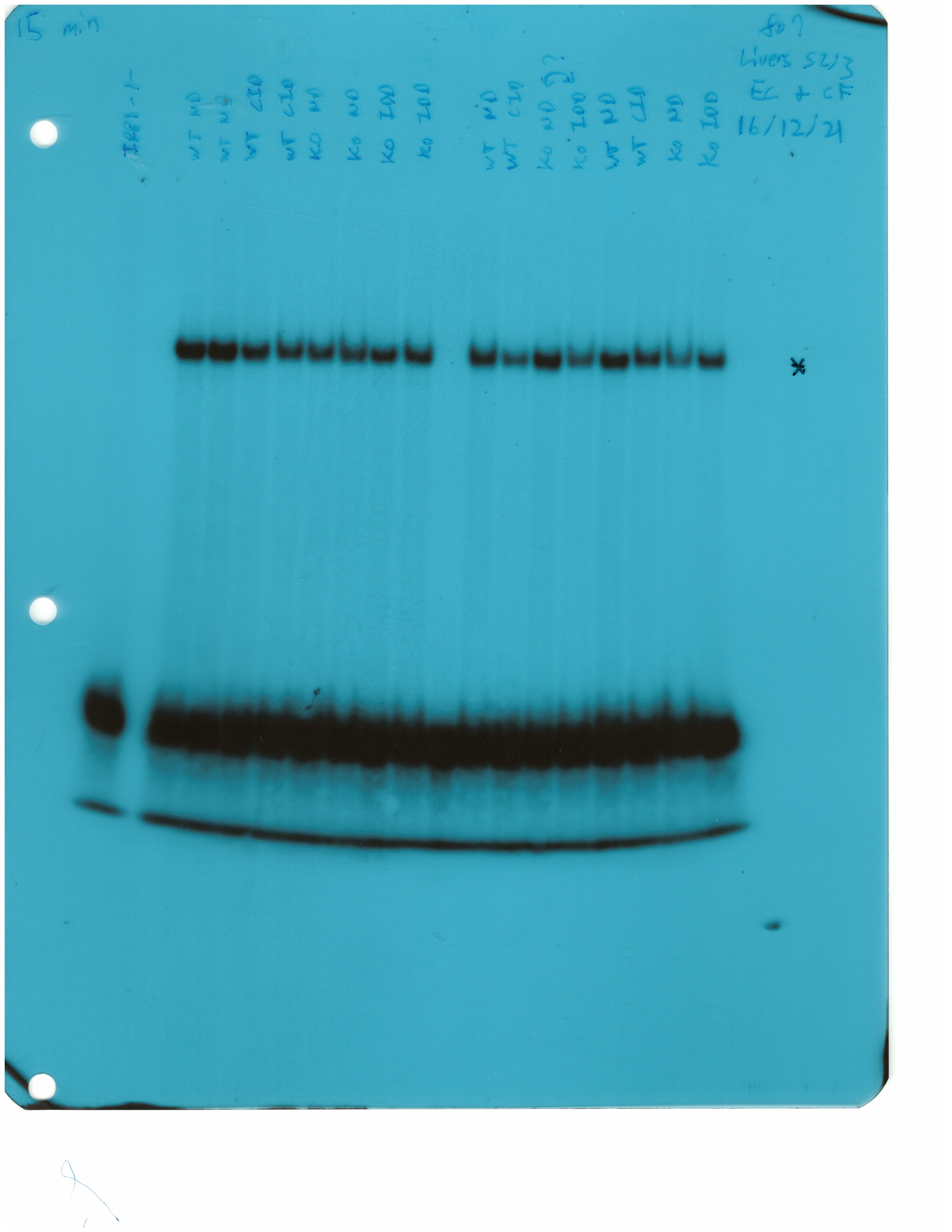

Supplement: Figure 6—source data 5. [file elife-81332-fig6-data5.jpg]

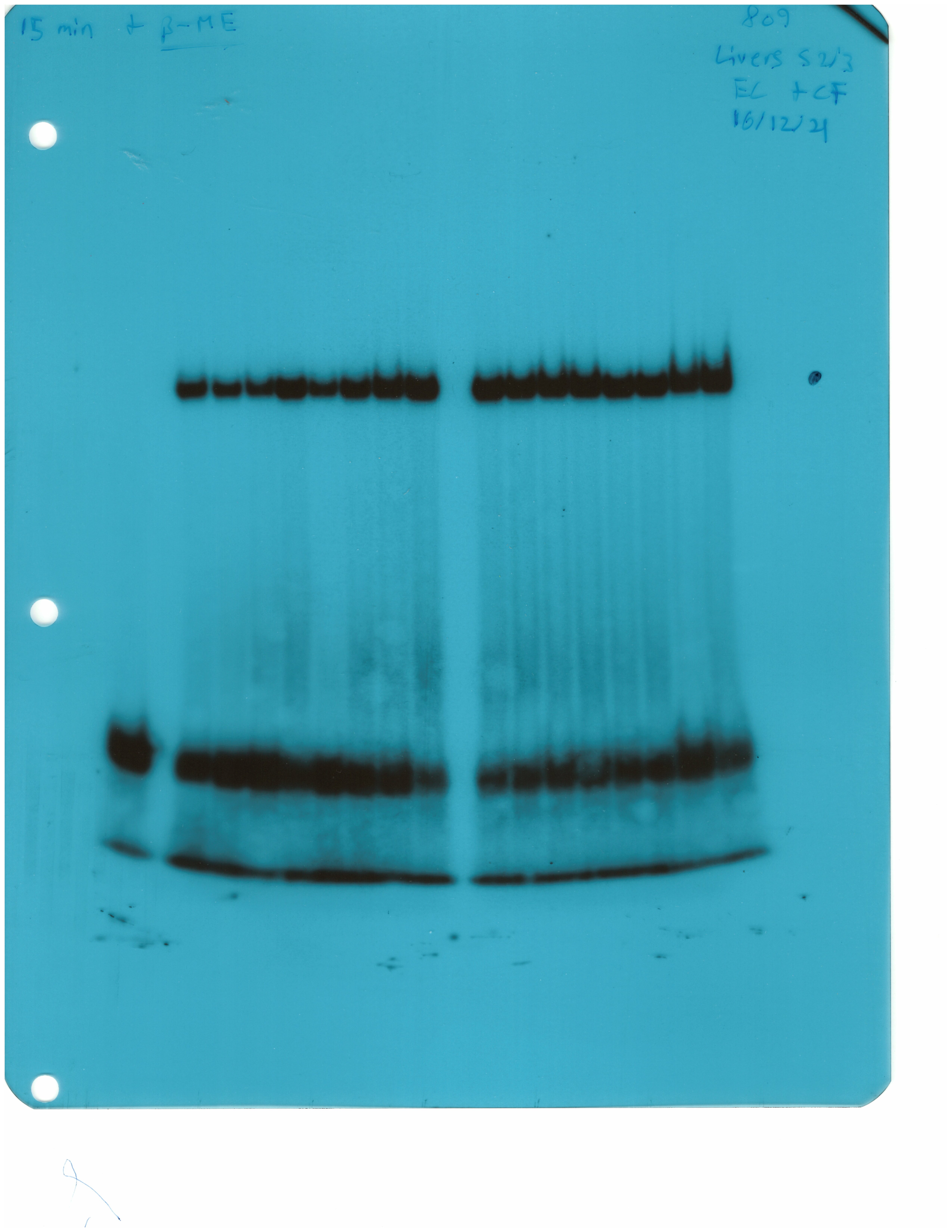

Supplement: Figure 6—source data 6. [file elife-81332-fig6-data6.jpg]

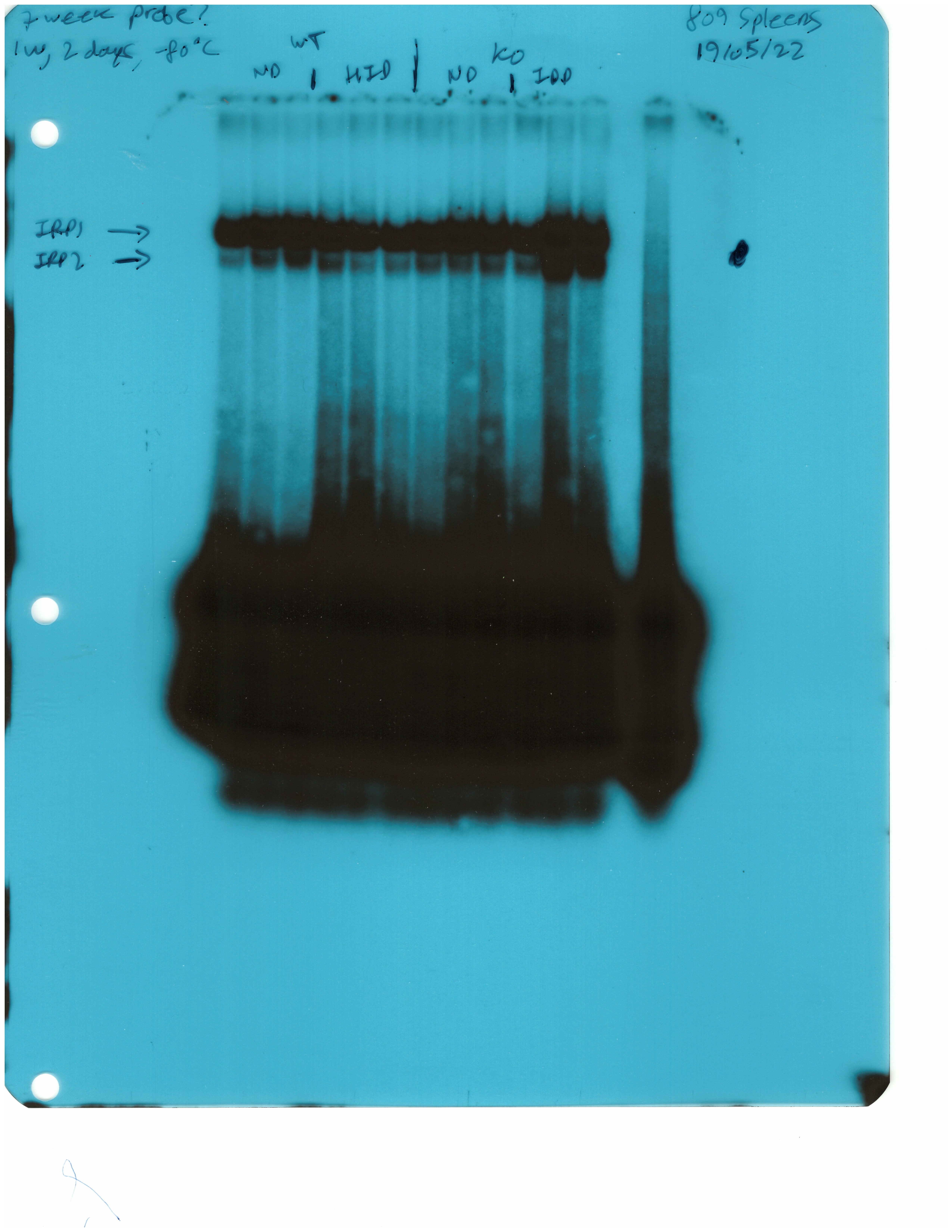

Supplement: Figure 6—source data 7. [file elife-81332-fig6-data7.jpg]

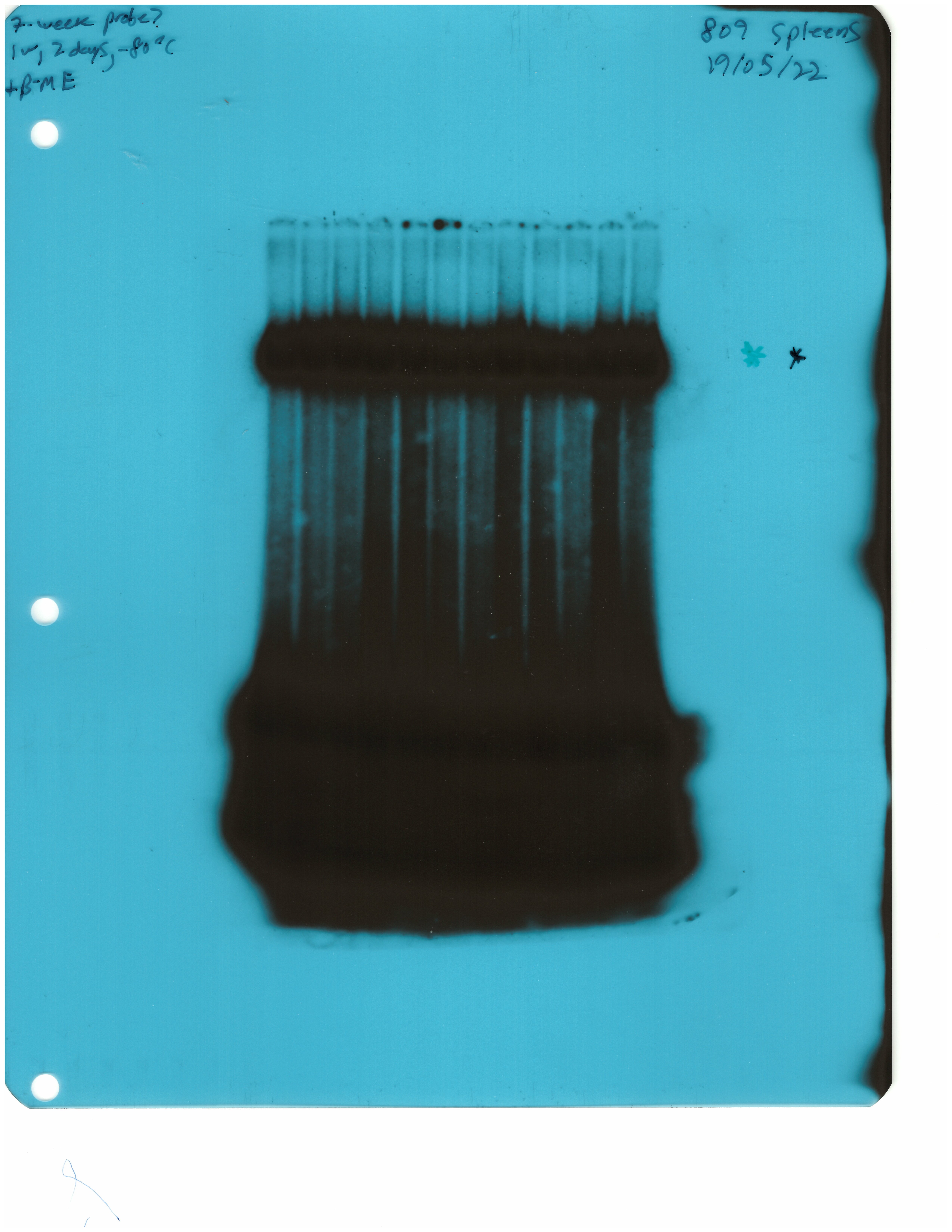

Supplement: Figure 6—source data 8. [file elife-81332-fig6-data8.jpg]

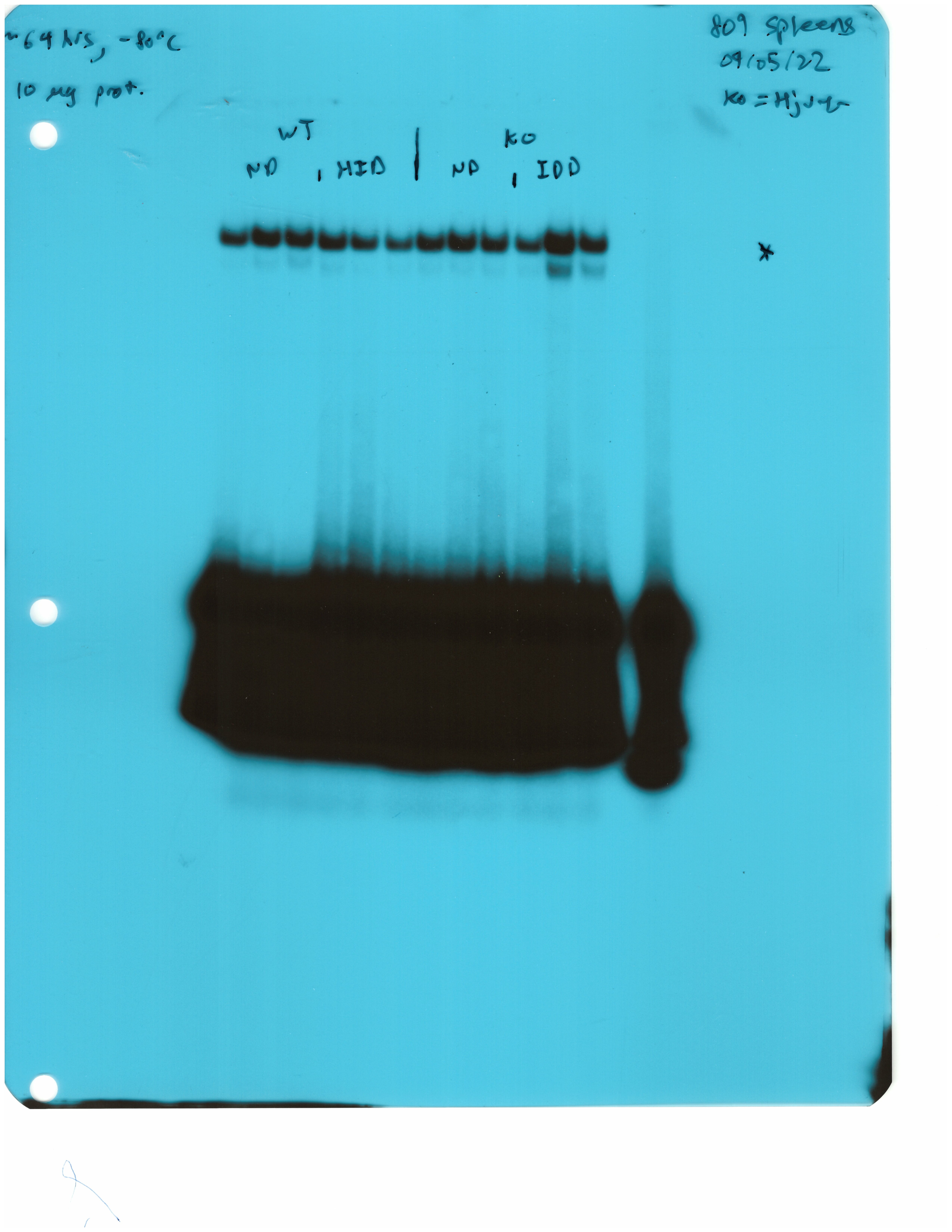

Supplement: Figure 6—source data 9. [file elife-81332-fig6-data9.jpg]

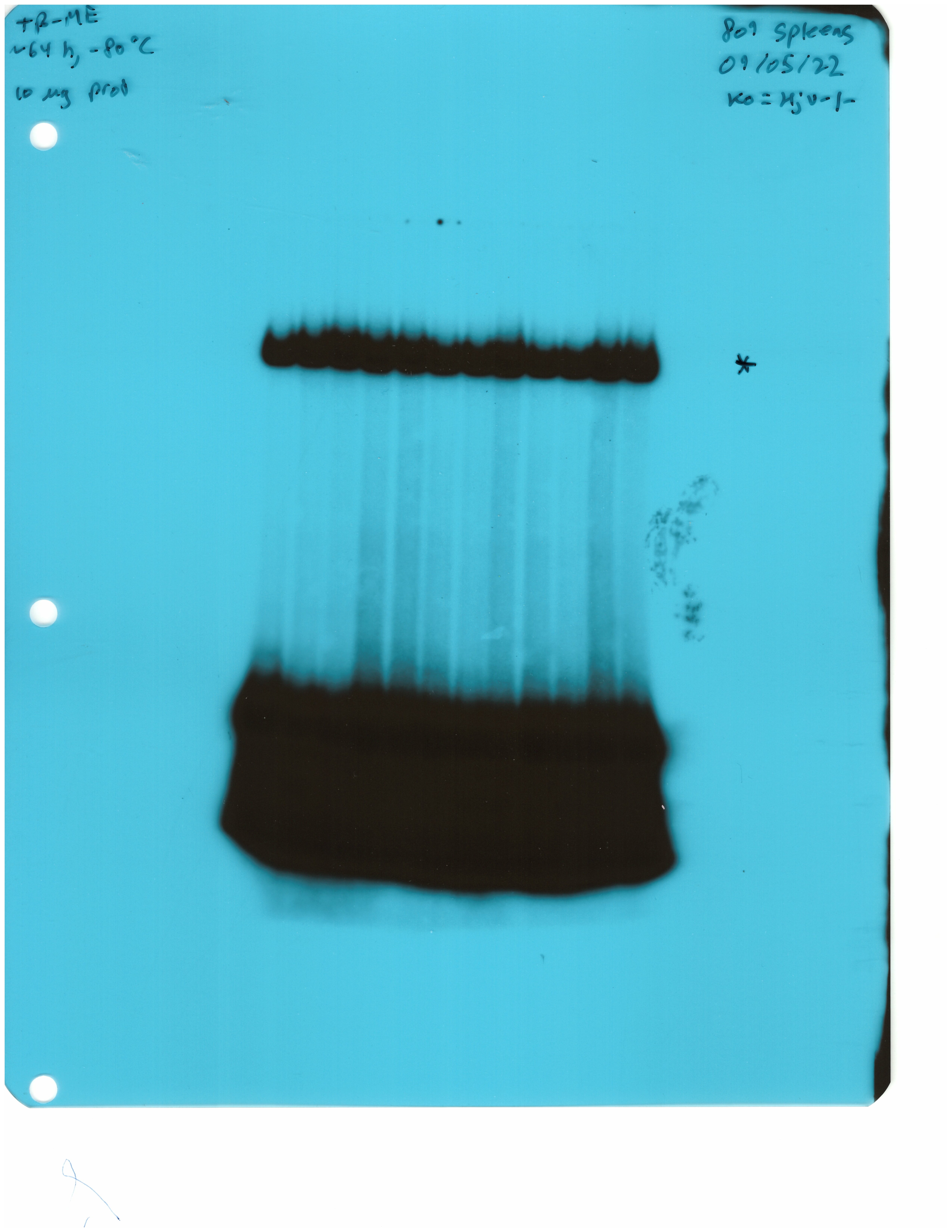

Supplement: Figure 6—source data 10. [file elife-81332-fig6-data10.jpg]

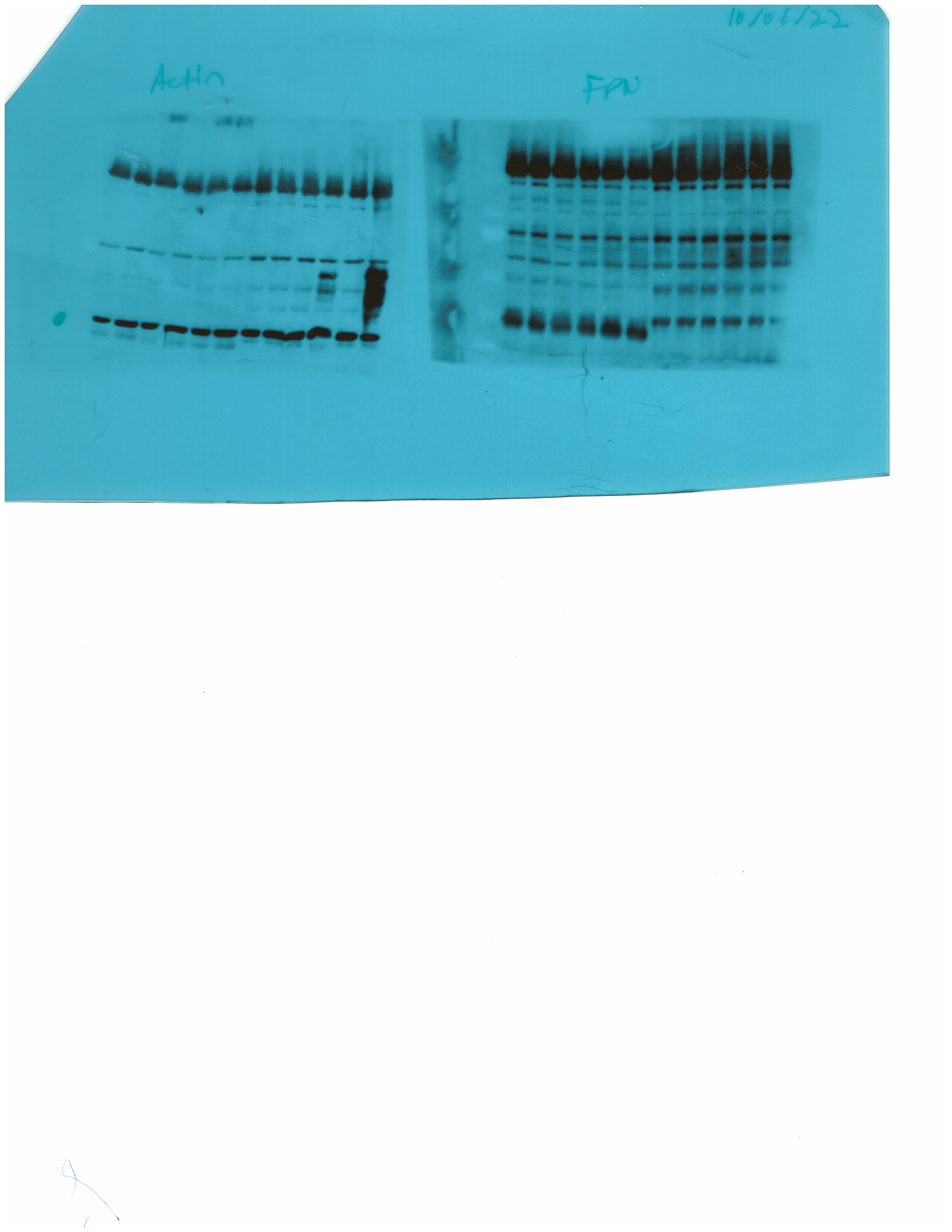

Supplement: Figure 6—source data 11. [file elife-81332-fig6-data11.jpg]

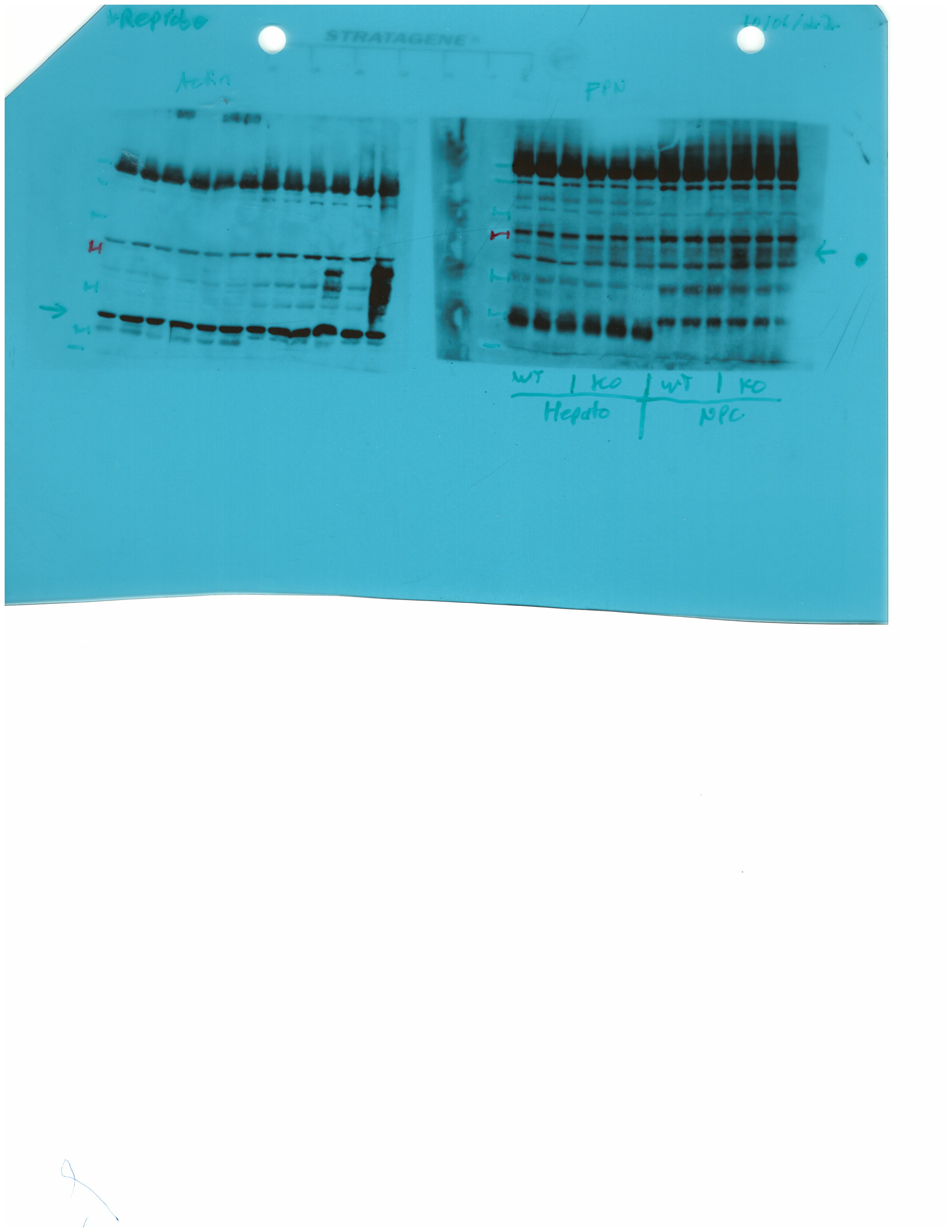

Supplement: Figure 6—source data 12. [file elife-81332-fig6-data12.jpg]

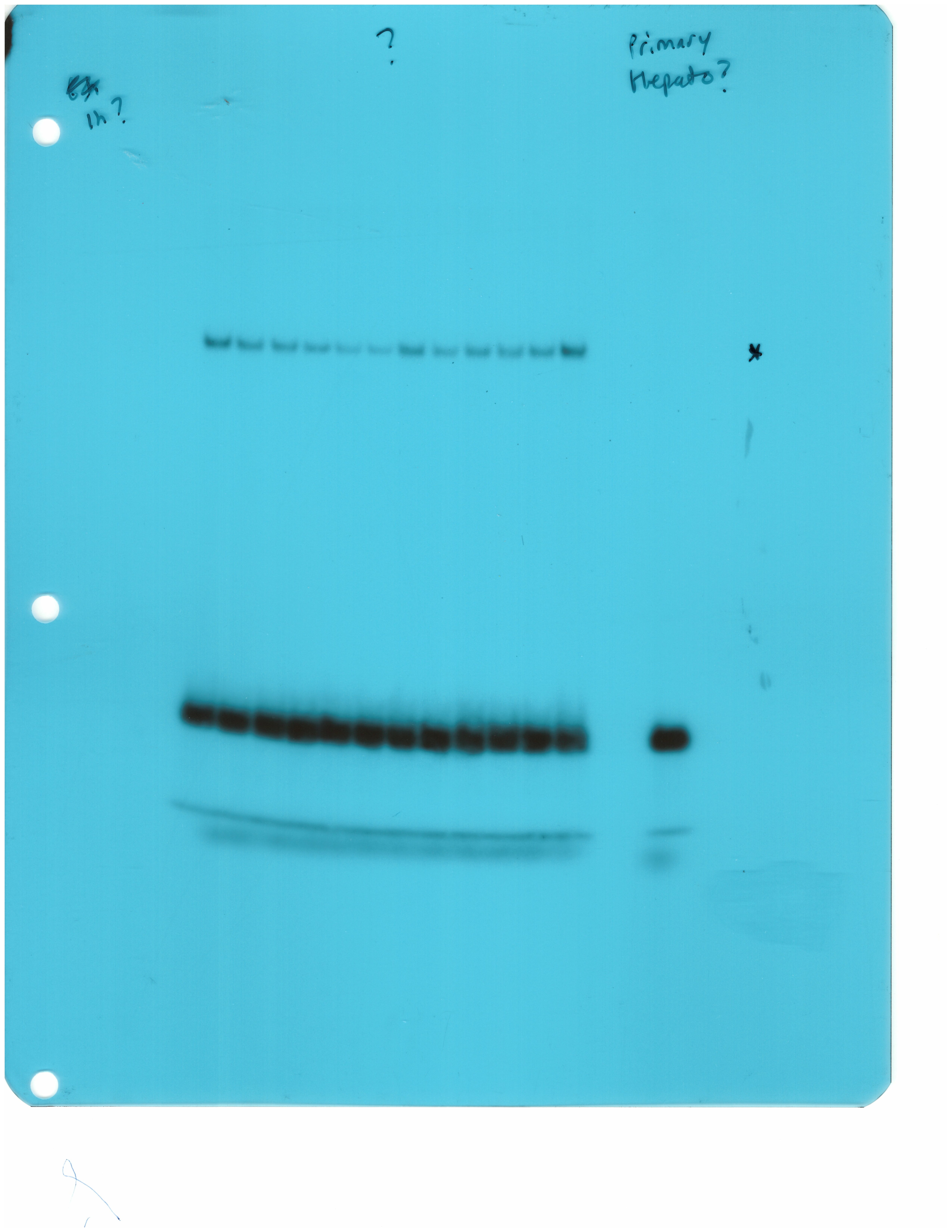

Supplement: Figure 6—source data 13. [file elife-81332-fig6-data13.jpg]

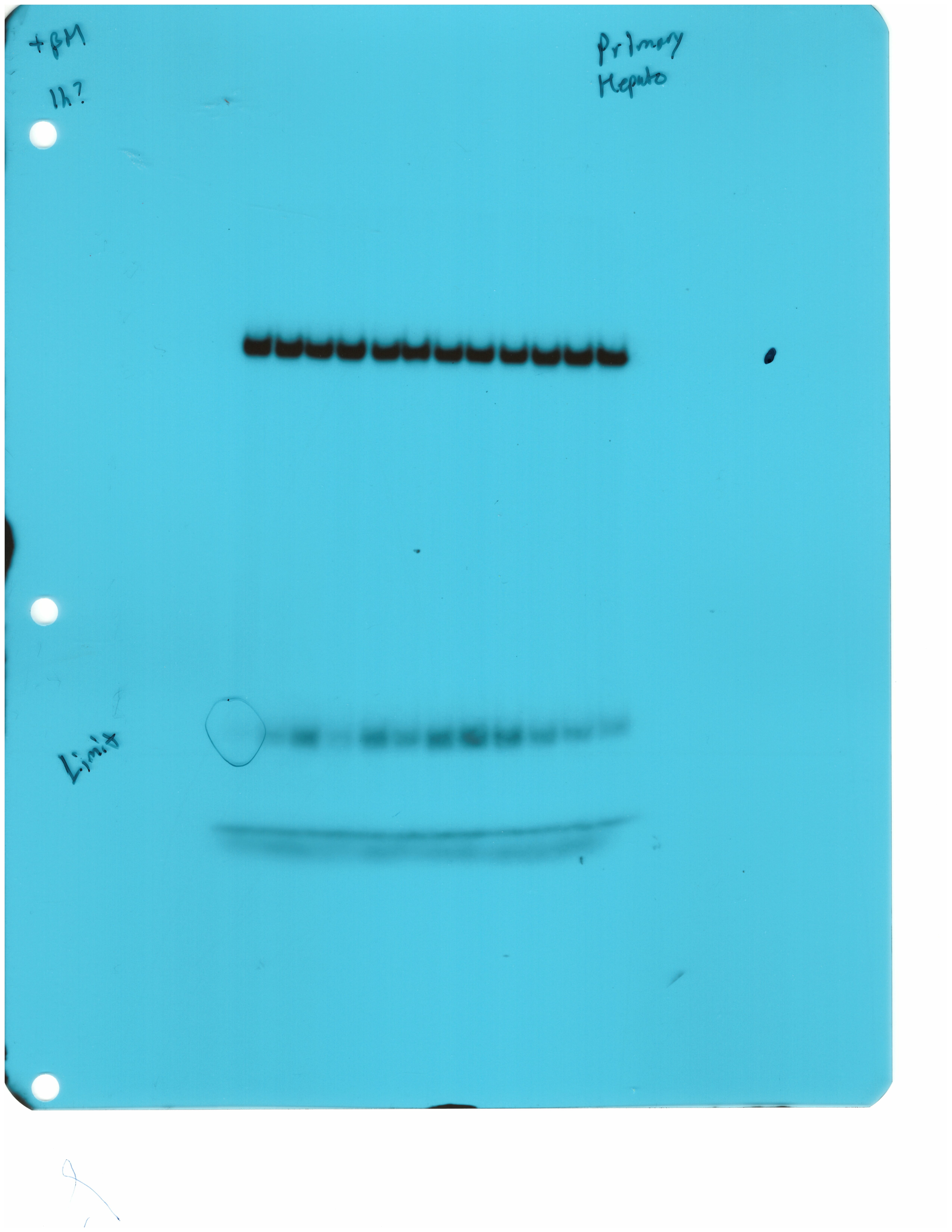

Supplement: Figure 6—source data 14. [file elife-81332-fig6-data14.jpg]

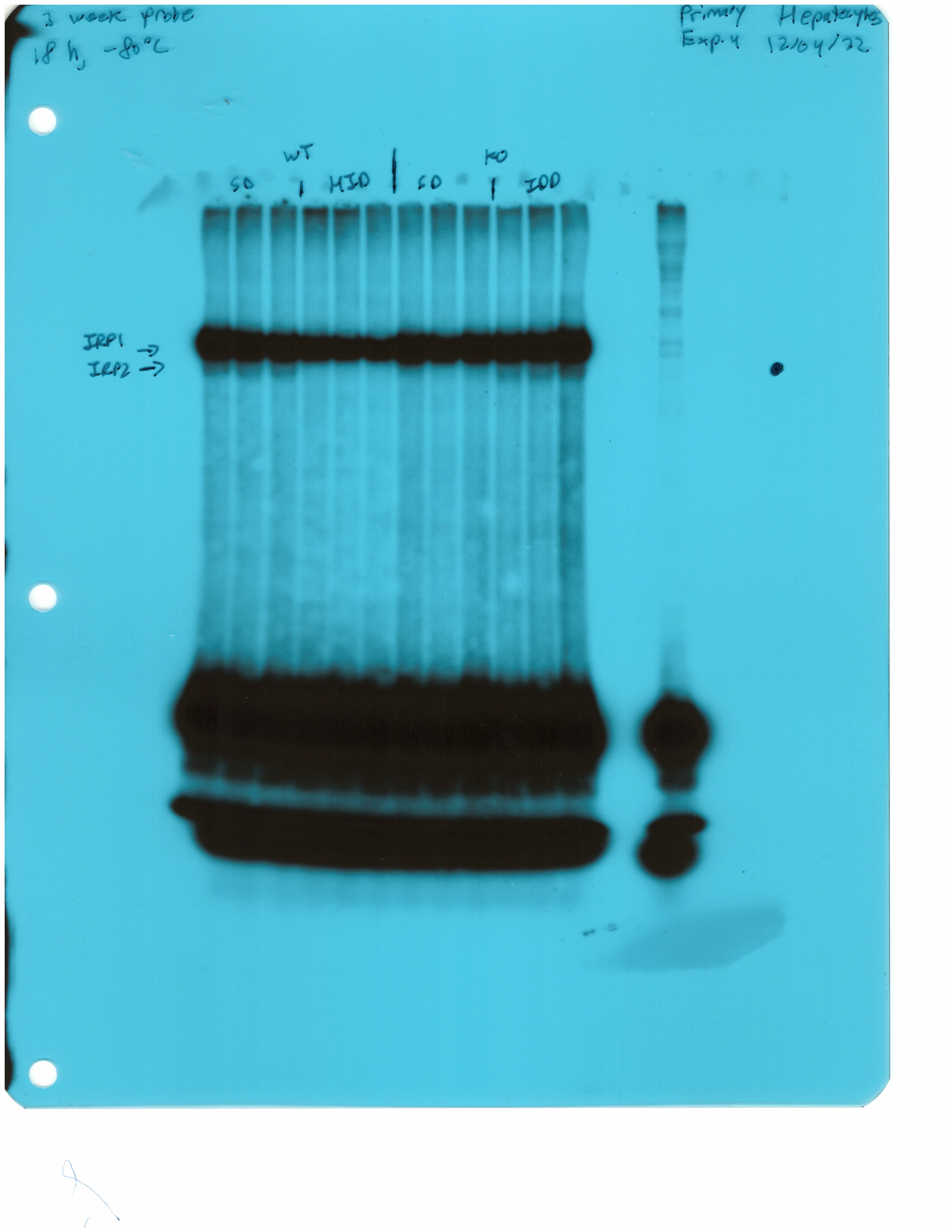

Supplement: Figure 6—source data 15. [file elife-81332-fig6-data15.jpg]

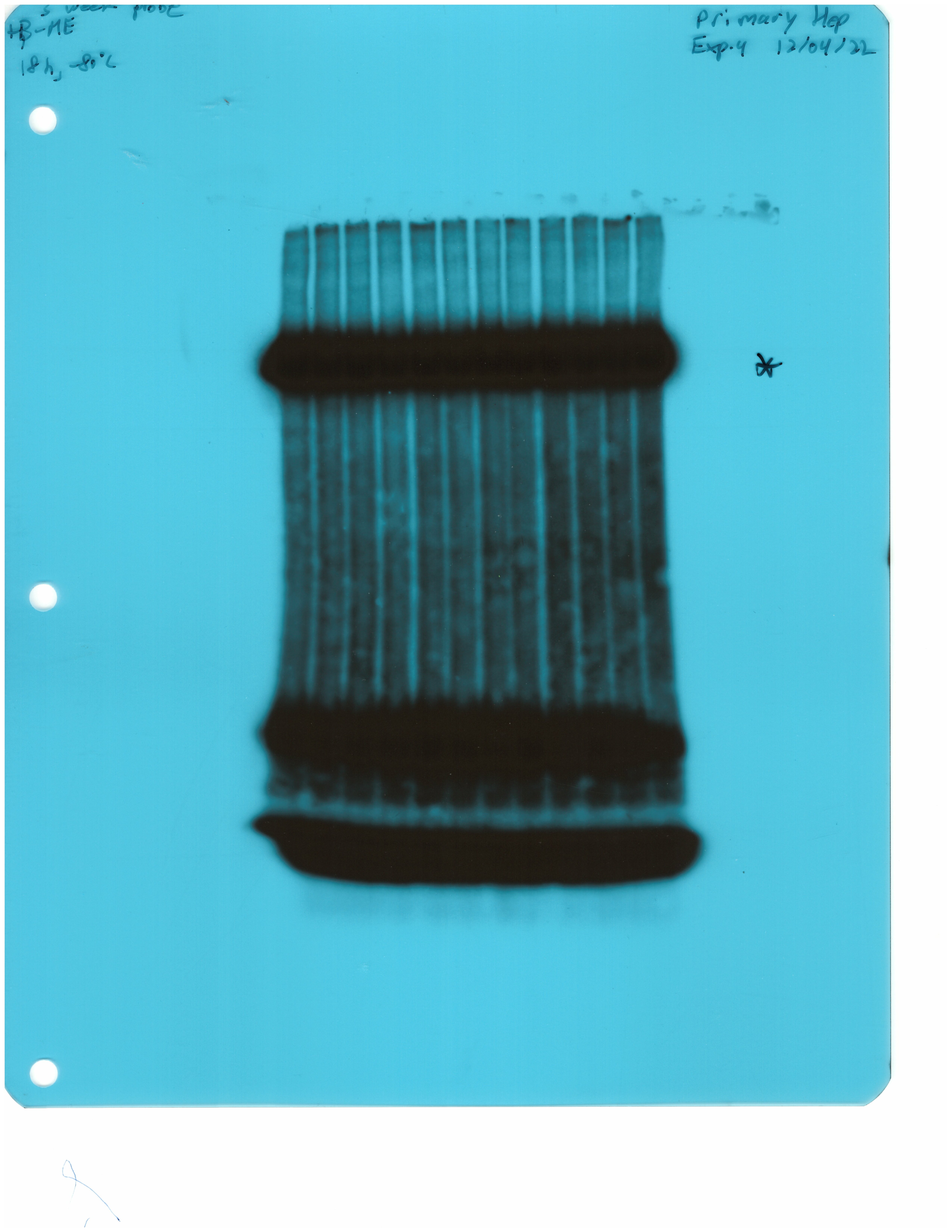

Supplement: Figure 6—source data 16. [file elife-81332-fig6-data16.jpg]

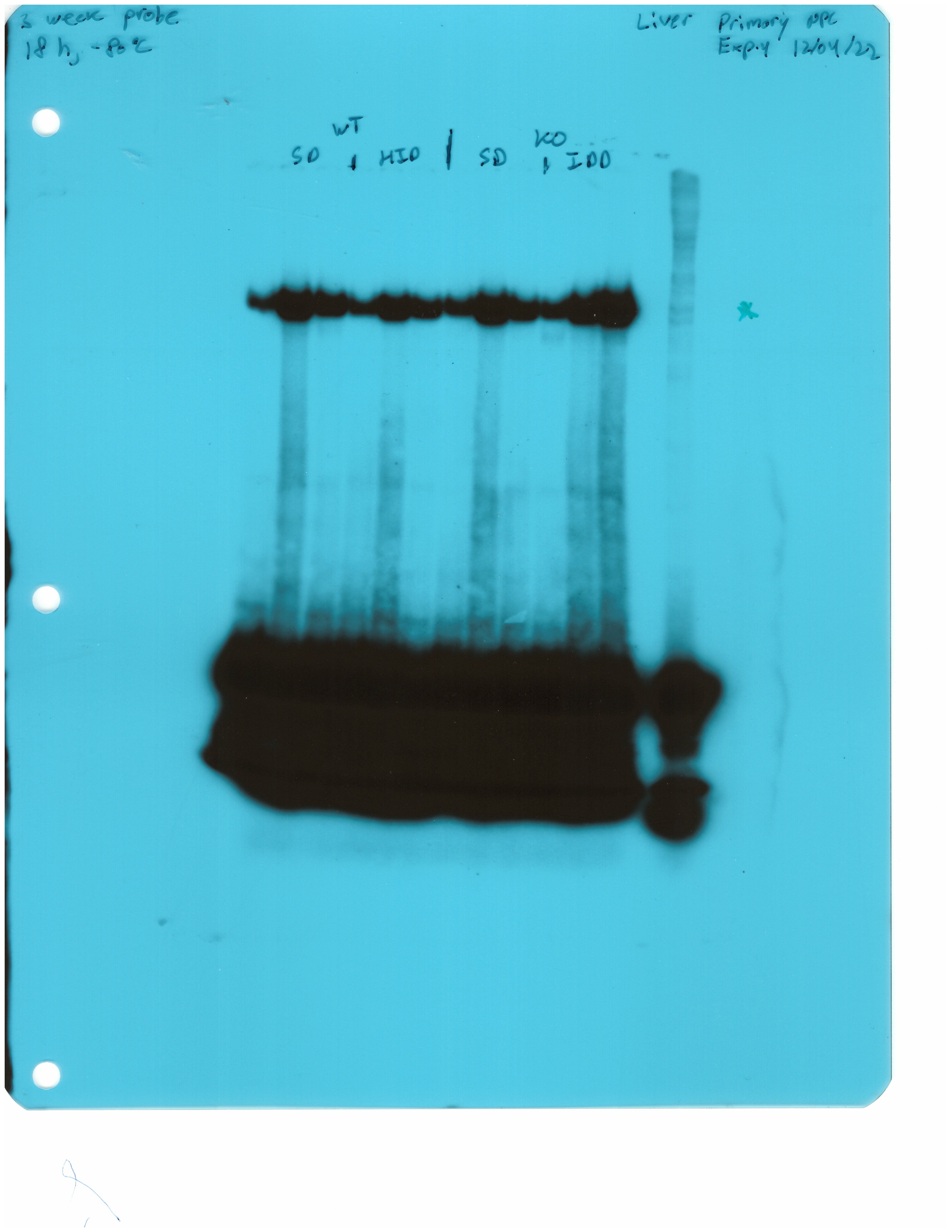

Supplement: Figure 6—source data 17. [file elife-81332-fig6-data17.jpg]

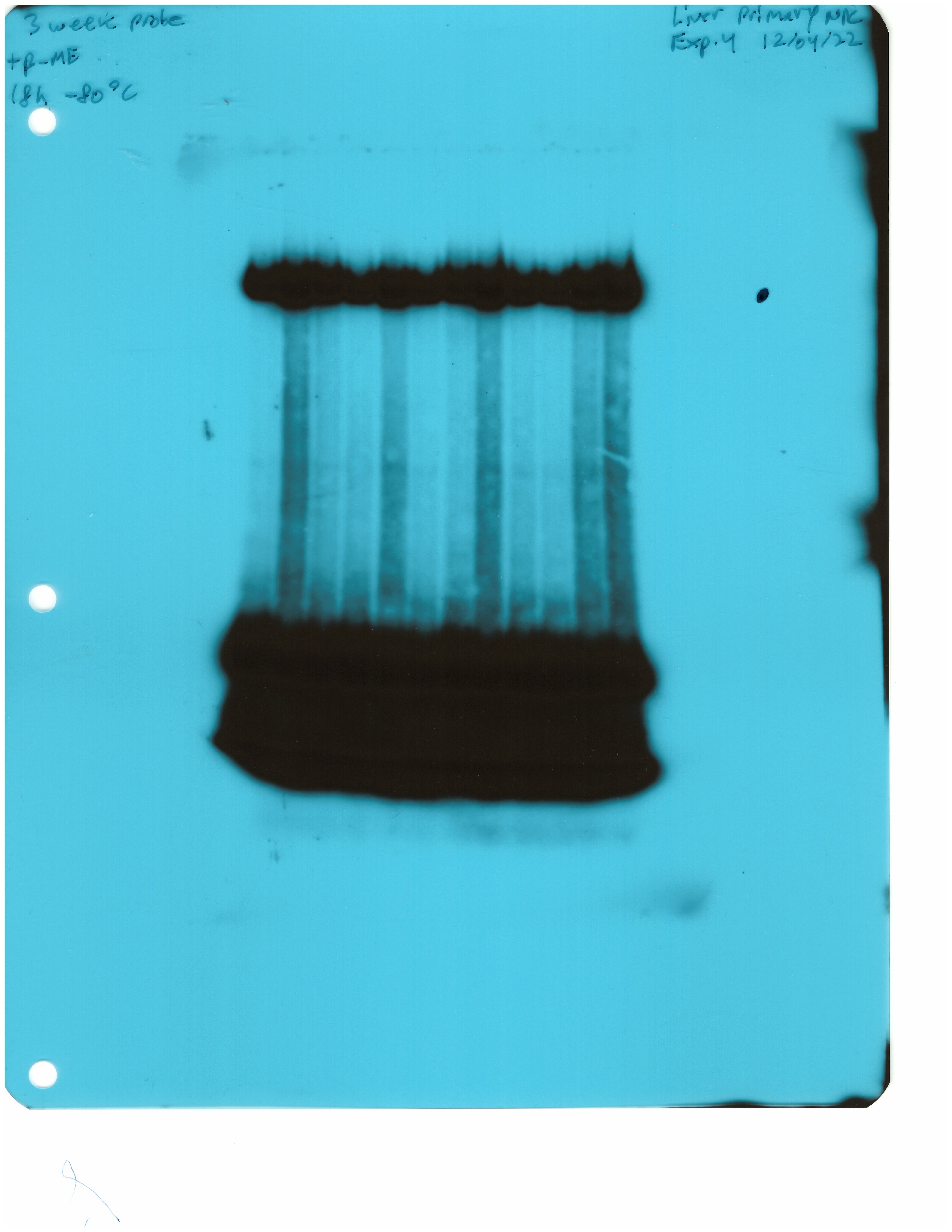

Supplement: Figure 6—source data 18. [file elife-81332-fig6-data18.jpg]

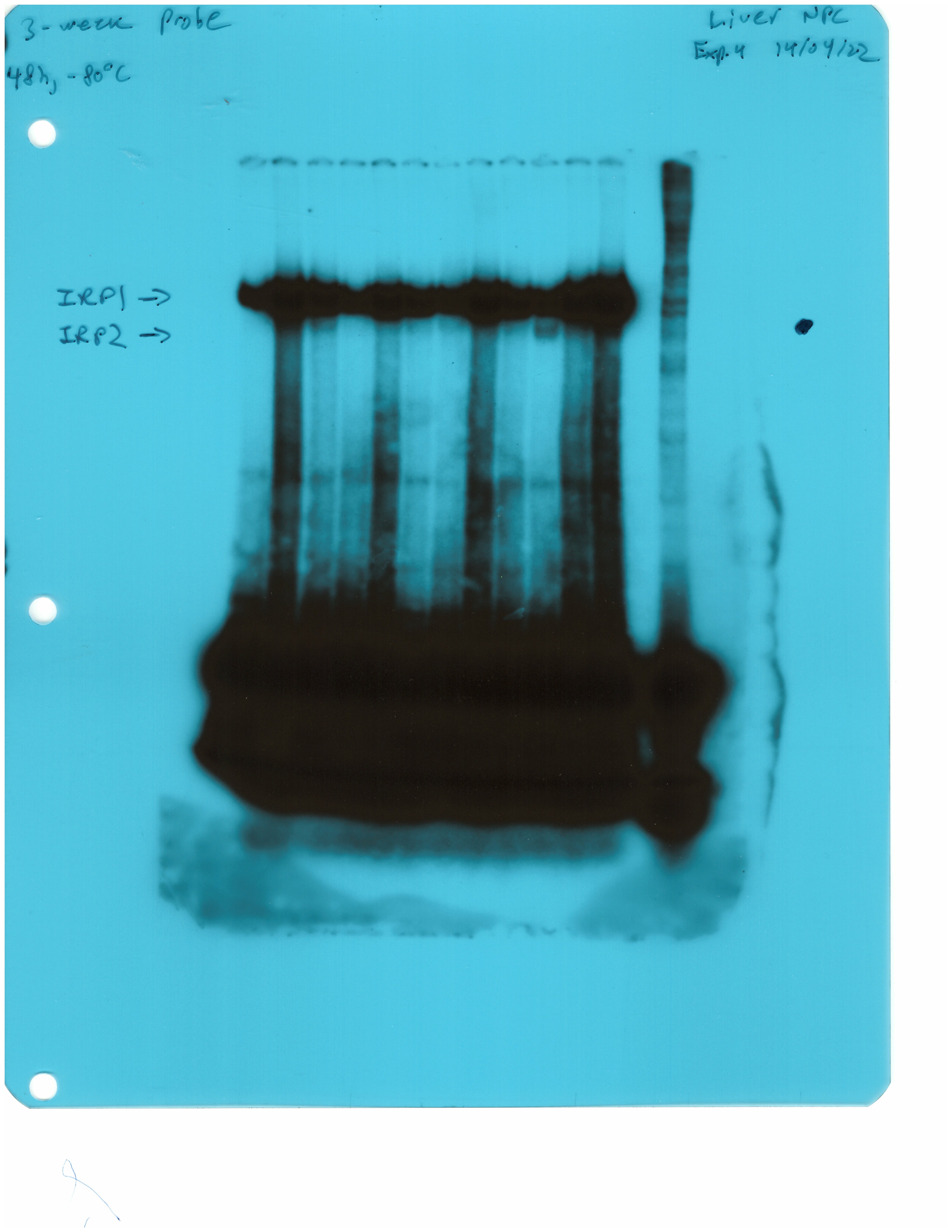

Supplement: Figure 6—source data 19. [file elife-81332-fig6-data19.jpg]

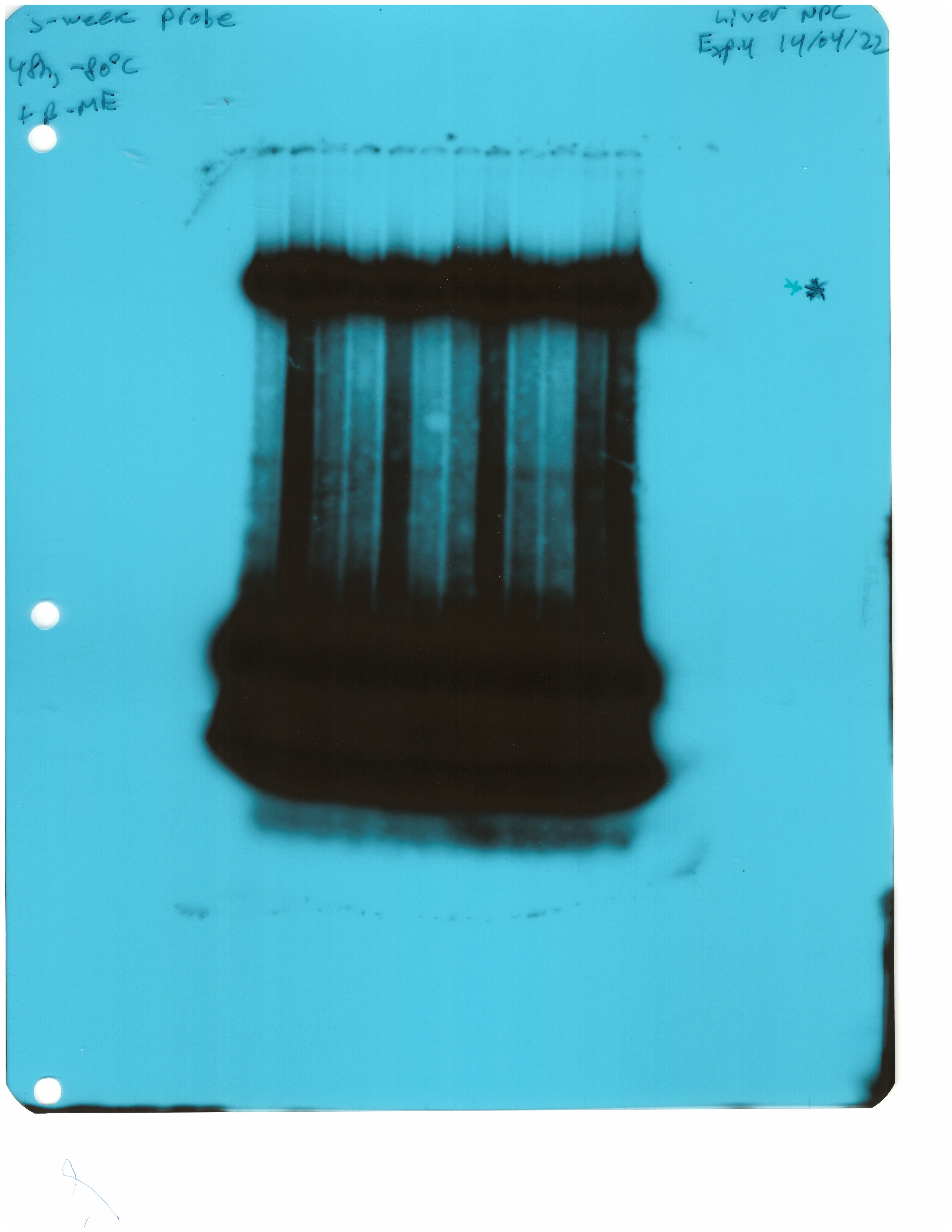

Supplement: Figure 6—source data 20. [file elife-81332-fig6-data20.jpg]
